# New Arylethanolimidazole Derivatives as HO-1 Inhibitors with Cytotoxicity against MCF-7 Breast Cancer Cells

**DOI:** 10.3390/ijms21061923

**Published:** 2020-03-11

**Authors:** Valeria Ciaffaglione, Sebastiano Intagliata, Valeria Pittalà, Agostino Marrazzo, Valeria Sorrenti, Luca Vanella, Antonio Rescifina, Giuseppe Floresta, Ameera Sultan, Khaled Greish, Loredana Salerno

**Affiliations:** 1 Department of Drug Sciences, University of Catania, viale A. Doria 6, 95125 Catania, Italy; valeria.ciaffaglione@phd.unict.it (V.C.); vpittala@unict.it (V.P.); marrazzo@unict.it (A.M.); sorrenti@unict.it (V.S.); lvanella@unict.it (L.V.); arescifina@unict.it (A.R.); giuseppe.floresta@unict.it (G.F.); 2 Consorzio Interuniversitario Nazionale di ricerca in Metodologie e Processi Innovativi di Sintesi (C.I.N.M.P.S.), Via E. Orabona, 4, 70125 Bari, Italy; 3 Institute of Pharmaceutical Science, King’s College London, Stamford Street, London SE1 9NH, UK; 4 Department of Molecular Medicine, College of Medicine and Medical Sciences, Princess Al-Jawhara Centre for Molecular Medicine, Arabian Gulf University, Manama 329, Bahrain; ameeraa@agu.edu.bh (A.S.); khaledfg@agu.edu.bh (K.G.)

**Keywords:** heme oxygenase, HO-1, imidazole, inhibitors, structure–activity relationships, anticancer

## Abstract

In this paper, a novel series of imidazole-based heme oxygenase-1 (HO-1) inhibitors is reported. These compounds were obtained by modifications of previously described high potent and selective arylethanolimidazoles. In particular, simplification of the central linker and repositioning of the hydrophobic portion were carried out. Results indicate that a hydroxyl group in the central region is crucial for the potency as well as the spatial distribution of the hydrophobic portion. Docking studies revealed a similar interaction of the classical HO-1 inhibitors with the active site of the protein. The most potent and selective compound (**5a**) was tested for its potential cytotoxic activity against hormone-sensitive and hormone-resistant breast cancer cell lines (MCF-7 and MDA-MB-231).

## 1. Introduction

Heme oxygenase HO is a family of heat-shock proteins that catalyzes the rate-limiting step of the heme degradative pathway. It is a stereospecific oxidative process, whose final products carbon monoxide (CO), ferrous iron (Fe^2+^), and biliverdin (BV), rapidly converted in bilirubin (BR) by biliverdin reductase, are generated in the equal amount [1]. Three different isoforms of HO have been identified until today: HO-1, HO-2, and HO-3, but only the first two possess enzymatic activity. HO-1 is the highly inducible isoform, which is detectable at basal conditions only in spleen, liver, and bone marrow [2]. In all other organs and tissues, the HO-1 amount increases following different stimuli able to induce HO-1 gene expression, especially oxidative stress, heavy metals, toxins, UV radiations, and xenobiotics [3]. The second isoform, HO-2, is constitutively present, mainly in brain and testis, where it plays neuroprotective roles, and it is involved in the development of germ cells [4]. Although HO-1 and HO-2 are encoded by two different genes (*HMOX1* and *HOMX2*, respectively), they share a 40% homology of the amino acid sequence [5]. HO system plays an essential cytoprotective role, firstly because it is responsible for heme degradation, avoiding the effects of the pro-oxidant free heme. Additionally, it leads to the production of beneficial catabolites, in particular CO, which is a gaseous transmitter that mediates anti-inflammatory, antiapoptotic, antiproliferative, and vasodilatory effects [6], while BV and BR counteract oxidant-mediated cell injury at nanomolar levels [7]. For these reasons, an increase of HO-1 expression and/or activity may be desirable in all those diseases with oxidative or inflammatory etiology, such as metabolic and cardiovascular diseases [8]. Indeed, several natural or synthetic compounds able to induce HO-1 have been studied for therapeutic application [9,10,11,12,13,14].

On the contrary abnormal HO-1 expression has been observed in some pathologies, contributing to the diffusion and maintenance of the illness. This scenario is particularly real in different types of human cancers [15,16]. Specifically, HO-1 overexpression can exert protective effects on cancer cells and may contribute to cancerogenesis, cancer progression and growth, metastasis, and, overall, development of resistance to chemo-, radio-, and photo-therapies [17]. In this regard, HO-1 inhibition may be viewed as a new opportunity for anticancer treatment [18,19,20,21]. Indeed, in recent years many HO-1 inhibitors have been described as anticancer agents, both for their intrinsic antiproliferative properties [22,23] or for their adjuvant effects in coadministration with antitumor drugs [24]. In this last case, HO-1 inhibitors may have synergistic activity with the anticancer drug, allowing a reduction of the dosage and the consequent side effects [25,26], or in some instances, they may help in overcoming resistance to anticancer therapy [20,21].

**Figure 1:**
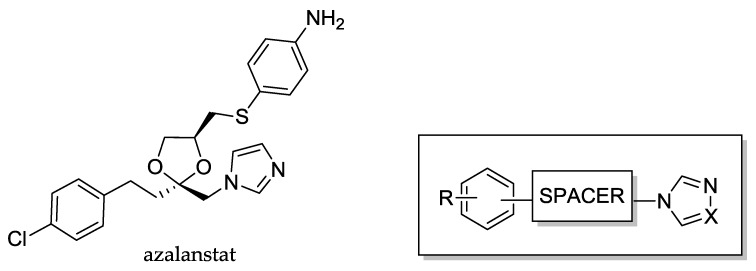
Azalanstat and simplified structure of HO-1 inhibitors.

Only two classes of HO-1 inhibitors have been described so far: (i) the metalloporphyrins, that are heme analogs, which bind competitively to the enzyme and usually do not discriminate from other heme-depending enzymes; (ii) the azole-based inhibitors, originated from structural modification of the first-discovered HO-1 inhibitor azalanstat (Figure 1). This last class of compounds shares a standard non-competitive binding mode and generally inhibits preferentially HO-1 with respect to other proteins. Structure–activity relationship (SAR) and crystallographic studies on the azole-based inhibitors have been performed in the last two decades; these studies afforded to the discovery of a restricted number of derivatives endowed with high potency for HO-1, and selectivity for the HO-1 over the HO-2 subtype. The results of these studies are extensively resumed in some recent reviews [27,28,29,30]. Most of the HO-1 inhibitors are characterized by an azole nucleus (i.e., imidazole or triazole) and a hydrophobic portion, connected by an alkyl or a heteroalkyl spacer, as in the simplified structure reported in Figure 1. 

**Figure 2:**
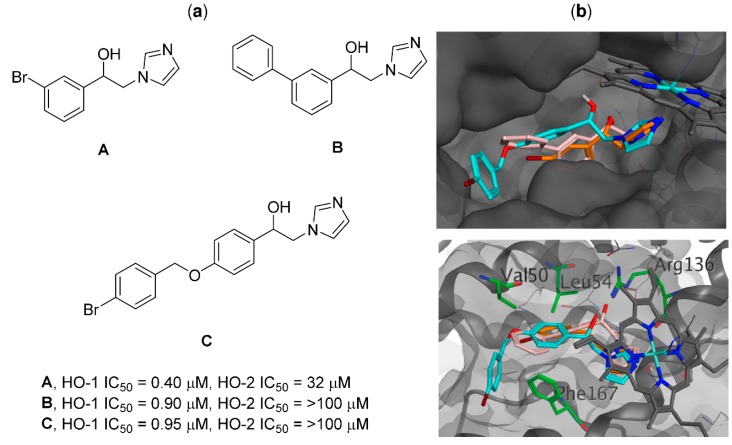
(**a**) Structures and HO IC_50_ values of reference HO-1 inhibitors **A**–**C** and (**b**) their binding with the enzyme [31].

Our group has been working in the field of HO-1 inhibitors for several years, contributing to the development of novel azole-based HO-1 inhibitors, which possess the classical chemical features required for the binding to the enzyme [22,25,31,32,33,34]. Among the discovered compounds, the class of arylethanolimidazoles stands out for its inhibitory properties, in particular compounds A–C (Figure 2a) were very potent and selective HO-1 inhibitors (HO-1 IC_50_ ranging from 0.4 to 0.9 μM, HO-2 IC_50_ ranging from 34 to > 100 μM). The best docking poses for compounds A–C are depicted in Figure 2b, describing the classical interactions with the pocket of HO-1, were the imidazole interacts with the iron of the heme group, and the aromatic portion is located in a hydrophobic region. Starting from A–C as reference compounds, in this work, we designed and synthesized a novel series of imidazole-based derivatives in which the connecting chain and the hydrophobic portion were further modified in order to optimize the observed activity. All the novel compounds **2a**–**c**, **5a**–**f**, **6a**,**b** were tested on HO-1 and the most potent also on HO-2. Docking studies also supported the results obtained from SAR studies. Finally, the most promising compound **5a,** which emerged from this new study [31,32] was investigated for its antitumor properties.

## 2. Results and Discussion

### 2.1. Chemistry

**Scheme 1:**
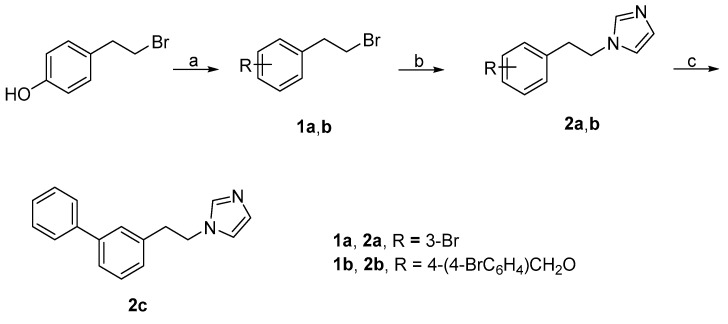
Reagents and conditions: (**a**) 4-bromobenzyl bromide, acetone, K_2_CO_3_, rt, 24 h; (**b**) imidazole, acetonitrile, TEA, TBAB, MW 90 °C, 45 min; and (**c**) phenylboronic acid, Pd(Ph_3_)_4_, toluene, ethanol, K_2_CO_3_, reflux.

The synthesis of compounds **2a**–**c** is described in Scheme 1. Phenethylbromide **1a** was commercially available, whereas the benzyloxy derivative **1b** was synthesized by etherification of 4-hydroxyphenethyl bromide with an excess of 4-bromobenzyl bromide, in acetone and K_2_CO_3_, at room temperature. *N*-alkylation of imidazole with the opportune phenethylbromides **1a**–**b** carried out in acetonitrile, in the presence of triethylamine (TEA) and tetrabutylammonium bromide (TBAB), under microwave irradiation, gave phenethylimidazoles **2a**,**b**. Compound **2c** was obtained through a Suzuki reaction between phenylboronic acid and the bromide derivative **2a**, in the presence of Pd(PPh_3_)_4_ and K_2_CO_3_ in toluene-ethanol under reflux.

**Scheme 2:**
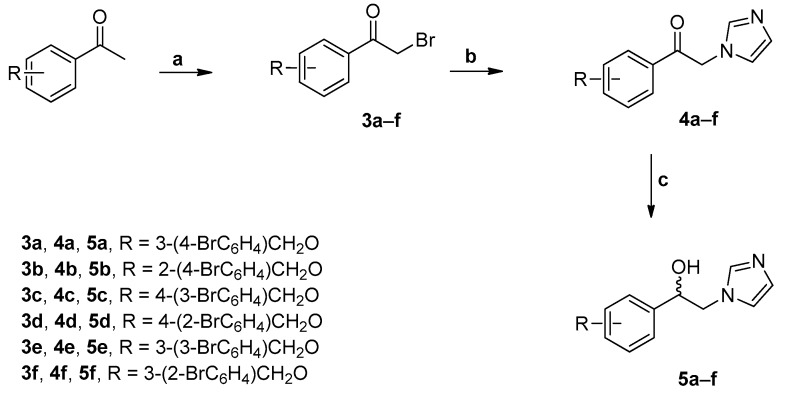
Reagents and conditions: (**a**) CuBr_2_, EtOAc/CHCl_3_, reflux, 5 h; (**b**) imidazole, K_2_CO_3_, DMF dry, rt, 2 h; and (**c**) NaBH_4_, methanol, reflux, 2 h.

The synthesis of compounds **5a**–**f** was accomplished in three steps, as shown in Scheme 2. Briefly, starting ketone derivatives, commercially available or prepared as previously reported [35,36,37], were brominated with CuBr_2_ in EtOAc/CHCl_3_ to obtain the 1-substituted bromomethyl ketones **3a**–**f**. Imidazole ketones **4a**–**f** were obtained through a nucleophilic displacement of **3a**–**f** using an excess of imidazole. Final ethanol derivatives **5a**–**f** were prepared in high yields reducing the corresponding ketones with NaBH_4_.

**Scheme 3:**
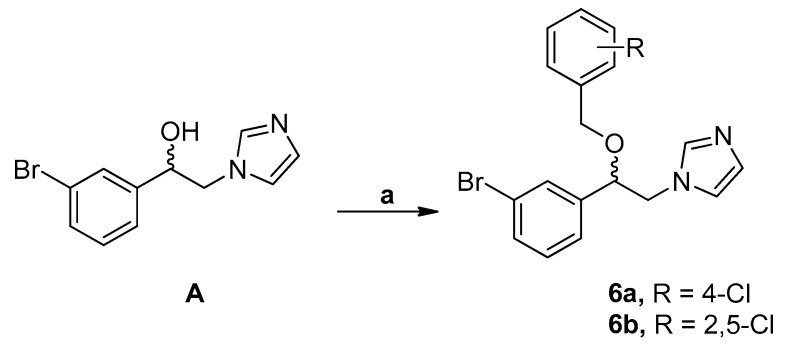
Reagents and conditions: (**a**) 1-(bromomethyl)-4-chlorobenzene/2-(bromomethyl)-1,4-dichlorobenzene, NaH, DMF dry, rt, 3 h.

Compounds **6a**,**b** were prepared by treatment of **A** in the presence of NaH and benzyl bromide in dry DMF, as shown in Scheme 3.

Final compounds **5a**–**f** and **6a**,**b** were obtained as racemic mixtures and tested without optical purification.

### 2.2. HO Inhibition and Structure–Activity Relationships (SARs)

In this work, all the novel synthesized compounds **2a**–**c**, **5a**–**f**, **6a**,**b**, as well as previously synthesized **4g**–**i**, were evaluated for inhibition of HO-1 activity; the most potent HO-1 inhibitors were also tested on HO-2. HO-1 and HO-2 were obtained from the microsomal fractions of rat spleen and rat brain, respectively. Enzymatic activity of both isoforms was determined by measuring the bilirubin formation using the difference in absorbance at 464–530 nm, as described in the experimental section. Compounds A–C, and tin protoporphyrin (SnPP) were used as reference substances. Inhibition of enzymes activity is expressed as IC_50_ (μM), and results are resumed in Table 1.

In designing compounds **2a**–**c**, **5a**–**f**, and **6a**,**b** we considered the results obtained in our previous study. Specifically, we recently found that many arylethanolimidazoles possessed outstanding inhibitory properties [32]. Among them, compounds A–C (Figure 2, Table 1) were the most potent and selective HO-1 inhibitors; then, they were selected as lead compounds (LCs) for a classical medicinal chemistry approach. Compounds **2a**–**c** and **4g**–**i** are the analogs of LCs in which the connecting ethanolic chain has been modified in an ethylene or ethanone one obtaining a chemical simplification, due to the lack of a chiral center. All of them showed a reduced capacity of inhibiting HO-1, with respect to all the reference substances indicating that reduction or oxidation of the hydroxyl group drastically reduced the inhibitors’ potency. Compounds **6a**,**b** are the benzyl analogs of LC **A**, and were designed in order to find additional contact points in the region of the enzyme that usually binds to the connecting chain of the inhibitors. This change of the ethanolic chain gave negative results, suggesting that also the substitution of the hydroxyl group reduced the inhibitors’ potency and confirming the crucial role of a hydroxyl group for the binding with the enzyme. Thus, we designed and synthesized compounds **5a**–**f**, which maintained the ethanolic spacer and were modified at the hydrophobic portion. It is well known that the hydrophobic portion of the inhibitors may be greatly modified since the western region of the enzyme is very flexible and can also accommodate large substituents [20,38]. Compounds **5a**,**b** are analogs of LC **C** in which the 4-bromobenzyloxy substituent was moved from the *para* to the *meta* or the *orto* position of the central phenyl ring. The *meta* analog **5a** maintained the same potency of reference compound **C** (**5a** HO-1 IC_50_ = 0.9 μM, **B** HO-1 IC_50_ = 0.95 μM) and is the most potent derivative of the whole series, whereas the *orto* analog **5b** was inactive (HO-1 IC_50_ ≥ 100 μM). Therefore, we finally synthesized derivatives **5c**–**f** where the bromine substituent was moved from the 4- to 3- or 2-position of the benzyloxy moiety. Among this subset of compounds, **5d** gave good results (HO-1 IC_50_ = 9 μM), whereas the other three analogs showed only moderate activity. These results indicate that the hydrophobic portion of these novel arylethanolimidazoles greatly influences the potency, both in terms of a steric hindrance than electronic distribution. Being compounds **5a** and **5d** the most potent HO-1 inhibitors of this series, they were also tested on HO-2. Derivative **5a** showed a good selectivity for HO-1/HO-2 (HO-2 IC_50_ = 54 μM), whereas **5d** was only mildly selective (HO-2 IC_50_ = 13.5 μM).

Taken together, these results suggest that the ideal structure should include both the hydroxyl group and the bromobenzyloxy substituent at the *para* or *meta* of the central phenyl ring. 

### 2.3. Docking Studies

**Figure 3:**
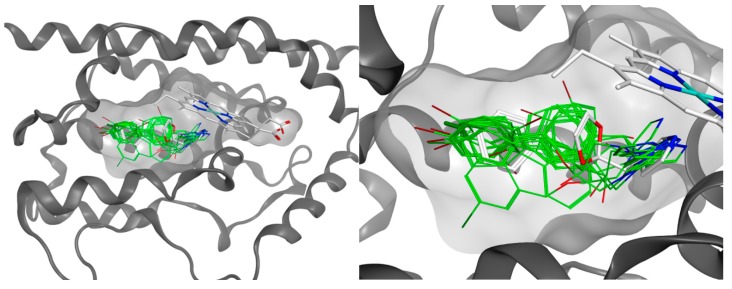
Binding poses of **2a**–**c**, **5a**–**f,** and **6a**,**b** (green) compared to the crystallized pose of QC-15 (white).

In order to figure out the most relevant interactions of the new molecules, we studied the binding of **2a**–**c**, **5a**–**f**, and **6a**,**b** employing the crystal structure of HO-1 complexed with QC-15 [39], through docking calculations. Docking was performed as reported in the experimental section. The results are collected in Table 2, and the docking poses are shown in Figure 3 and Figures S23–S25. 2D representations of each binding pose are reported in Figures S26–S36. The calculated binding energies (calculated *K*_i_) are in good accordance with the experimental measured IC_50_ values in the HO-1 inhibition assay. All of the studied molecules present the nitrogen atom of the imidazole ring located in the eastern region of the HO-1 pocket in the proximity of the ferrous iron of heme, as normally expected from such class of compounds. Utilizing this geometry, the iron (II) is coordinated to the nitrogen of the imidazole ring, and it is protected from oxidation by breakage of an ordered solvent structure involving the crucial Asp140 hydrogen-bond network (Tyr58, Tyr114, Arg136, and Asn210) and the consequent shift of different water molecules required for the catalytical activity of the protein. The three different classes of studied compounds (**2**, **5**, and **6**) have a similar interaction with the binding pocket of HO-1, as reported in Figures S23–S25. In all the docked structures, the aromatic portions of the ligands are always located in the western region of the binding pocket. In particular, compound **5a** results as the most potent compound of the series, having an IC_50_ in the low micromolar range; the docking pose of this molecule revealed interactions similar to the classical HO-1 inhibitors, where the aromatic group is in the principal western region pocket (Phe166, Phe167, Val50, Phe37, and Leu147) and the bromine atom is located deep inside in the pocket. Differently to similar molecules in the same series, the geometry of molecule **5a** allows the first aromatic ring to stay in a different position compared to the same ring in the other molecule of the series. In this way, **5a** has optimum contact with the binding pocket residues and reaches the highest binding energy value. However, with the docking experiments, it was verified that the secondary aromatic group of the branched molecules **6** is not located inside the northeastern region, but it prefers to stay in the secondary western pocket. This could be an advantage for future designed molecules based on the same structure of **6**, considering that it was concluded that modification in the northeastern region would result in neither potency nor selectivity increases and may not be an efficient avenue in the development of highly selective HO-1 inhibitors.

### 2.4. In Vitro Cytotoxic Activity

**Figure 4:**
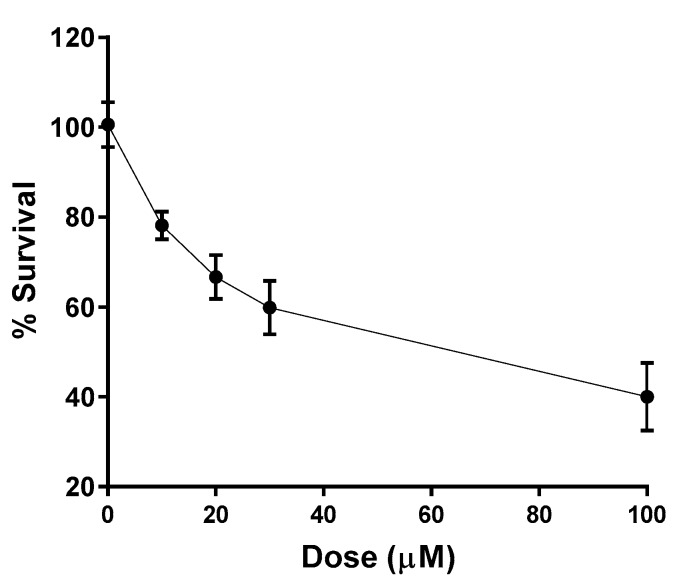
Cytotoxicity of compound **5a** against MCF-7 cell line (hormone-sensitive breast cancer cells). Data are expressed as mean ± SEM (*n* = 3). Nonlinear regression and IC_50_ value determination were performed using GraphPad Prism 6.

Compound **5a**, resulting in the most potent and selective analog within this series, was selected to evaluate its cytotoxic activity on hormone-sensitive (MCF-7) and hormone-resistant (MDA-MB-231) breast cancer cell lines. Both cell lines were treated with 10–100 μM of compound **5a** for 48 h. Cell survival at different concentrations was calculated compared to untreated controls. At the end of treatment, cell number was determined using the sulforhodamine B (SRB) colorimetric assay based on the measurement of cellular protein content. The tested compound exerted only moderate cytotoxic effects (IC_50_ = 47.36 ± 6.8 µM) towards the MCF-7 sensitive cells (Figure 4), whereas no cytotoxicity against MDA-MB-231 resistant cells was observed.

## 3. Materials and Methods

### 3.1. Chemistry

Melting points were determined by using an Electrothermal IA9200 apparatus containing a digital thermometer. Determinations were achieved after introducing glass capillary tubes, filled with analytes, inside the apparatus, and are uncorrected. Elemental analyses for C, H, N, and O were within ± 0.4% of theoretical values and were accomplished through a Carlo Erba Elemental Analyzer Mod. 1108 apparatus (Table S1). ^1^H NMR and ^13^C NMR spectra were recorded on Varian Inova Unity (200 and 500 MHz) spectrometers in DMSO-*d*_6_ solution (Figures S1–S22, supplementary materials). Chemical shifts are given in *δ* values to two digits after the decimal point in part per million (ppm), using tetramethylsilane (TMS) as the internal standard; coupling constants (*J*) are given in Hz. Signal multiplicities are indicated with the following abbreviations: s (singlet), d (doublet), t (triplet), q (quartet), m (multiplet), and br (broad signal). Reactions were monitored by thin-layer chromatography (TLC), carried out on Merck plates (Kieselgel 60 F254), using UV light (254 and 366 nm) for visualization and developed using iodine chamber. Flash column chromatography was performed on Merck silica gel 60 0.040‒0.063 mm (230‒400 mesh). Automated column chromatography was also done using a Biotage FlashMaster Personal Plus system with prepacked silica gel columns of different sizes (Biotage^®^ SNAP cartridge KP-Sil, Uppsala, Sweden). Where indicated, Celite^®^ was used as a filter aid. Synthetic procedures achieved through microwaves were performed with a CEM Discover instrument using closed Pyrex glass tubes (ca. 10 mL) with Teflon-coated septa. Reagents, solvents, and starting materials were purchased from commercial suppliers.

#### Synthesis of 1-bromo-4-((4-(2-bromoethyl)phenoxy)methyl)benzene (**1b**)

4-hydroxyphenethyl bromide (4.9 mmol) was dissolved in acetone (20 mL); K_2_CO_3_ (10 mmol) and the 4-bromobenzyl bromide (9.9 mmol) were added. The reaction mixture was left stirring for 24 h at rt. The solvent was evaporated under vacuum, and water was added. The obtained white solid was filtered under vacuum and washed with water until neutrality. Recrystallization with methanol gave the pure compound. White solid; mp 110.5–112.5 °C; yield 54%. ^1^H NMR (200 MHz, DMSO-*d*_6_): 7.60–7.56 (m, 2H, aromatic), 7.42–7.38 (m, 2H, aromatic), 7.21–7.17 (m, 2H, aromatic), 6.95–6.91 (m, 2H, aromatic), 5.06 (s, 2H, CH_2_O), 3.67 (t, *J* = 7.2 Hz, 2H, CH_2_), 3.04 (t, *J* = 7.2 Hz, 2H, CH_2_).

### 3.2. General Procedure for the Synthesis of Phenylethylimidazole Derivatives (***2a**,**b***)

To a mixture of the appropriate bromoethylbenzene **1a**,**b** (1.9 mmol) in acetonitrile (3 mL), imidazole (2.8 mmol), TEA (1.9 mmol), and TBAB (0.1 g, catalytic) were added. The reaction mixture was stirred for 45 min under microwave irradiation in a sealed vial (90 °C, 150 W, 150 Psi). The solvent was evaporated under vacuum, water, and NaOH 0.1 N were added and the resulted aqueous layer was extracted with EtOAc (3 × 50 mL). The organic layer was dried over anhydrous Na_2_SO_4_, filtered, and concentrated. The obtained residue was purified using a Biotage^®^ chromatographic system with Biotage^®^ SNAP KP-Sil flash chromatography cartridges and 9.5 EtOAc:0.5 MeOH as eluent.

#### 3.2.1. 1-(3-bromophenethyl)-1H-imidazole (**2a**)

Colorless oil; yield 57%. ^1^H NMR (500 MHz, DMSO-*d*_6_): 7.50 (s, 1H, imidazole), 7.38 (s, 2H, aromatic), 7.22 (t, *J* = 8.0 Hz, 1H, aromatic), 7.18–7.11 (m, 1H aromatic + 1H imidazole), 6.84 (s, 1H, imidazole), 4.19 (t, *J* = 7.2 Hz, 2H, CH*_A_*CH_B_), 3.01 (t, *J* = 7.2 Hz, 2H, CH_A_CH*_B_*). ^13^C NMR (125 MHz, DMSO-*d*_6_) 141.24, 137.38, 131.64, 130.69, 129.57, 128.34, 128.02, 121.83, 119.50, 47.07, 36.37. Anal. Calcd. for (C_11_H_11_BrN_2_): C, 52.61; H, 4.42; N, 11.16. Found: C, 52.69; H, 4.48; N, 11.19.

#### 3.2.2. 1-(4-((4-bromobenzyl)oxy)phenethyl)-1H-imidazole (**2b**)

White solid; mp 147.5–149.7 °C; yield 70%. ^1^H NMR (500 MHz, DMSO-*d*_6_): 7.58 (d, *J* = 8.3 Hz, 2H, aromatic), 7.48 (s, 1H, imidazole), 7.39 (d, *J* = 8.3 Hz, 1H, aromatic), 7.12 (s, 1H, imidazole), 7.08 (d, *J* = 8.6 Hz, 2H, aromatic), 6.90 (d, *J* = 8.6 Hz, 2H, aromatic), 6.84 (s, 1H, imidazole), 5.04 (s, 2H, CH_2_O), 4.15 (t, *J* = 7.3 Hz, 2H, CH*_A_*CH_B_), 2.94 (t, *J* = 7.3 Hz, 2H, CH_A_CH*_B_*). ^13^C NMR (125 MHz, DMSO-*d*_6_) 156.77, 137.16, 136.68, 131.34, 130.60, 129.71, 128.20, 120.88, 119.20, 114.72, 68.36, 47.41, 35.91. Anal. Calcd. for (C_18_H_17_BrN_2_O_2_): C, 60.52; H, 4.80; N, 7.84. Found: C, 60.48; H, 4.86; N, 7.80.

#### 3.2.3. Synthesis of 1-(2-((1,1’-biphenyl)-3-yl)ethyl)-1H-imidazole (**2c**)

Compound **2a** (1.2 mmol) was dissolved in toluene (10 mL) and EtOH (1 mL). Phenylboronic acid (1.8 mmol) and K_2_CO_3_ (3.5 mmol in aqueous solution 2M) were added under a nitrogen atmosphere and left stirring for 30 min. After that, Pd(Ph_3_)_4_ (5%) was added. The reaction mixture was left refluxing for 30 h under nitrogen atmosphere. DCM was added, and the mixture was filtered through a celite pad. The solvent was evaporated under vacuum, and diethyl ether (100 mL) was added to the residue. The resulted organic layer was washed with water and brine, dried over anhydrous Na_2_SO_4_, filtered, and concentrated. The obtained residue was purified using a Biotage^®^ chromatographic system with Biotage^®^ SNAP KP-Sil flash chromatography cartridges and 9.5 EtOAc:0.5 MeOH as eluent. Yellow oil; yield 27%. ^1^H NMR (500 MHz, DMSO-d_6_): 7.60 (d, J = 7.3 Hz, 2H, aromatic), 7.52 (s, 1H, imidazole), 7.51–7.42 (m, 3H, aromatic), 7.40 (s, 1H, imidazole), 7.35 (td, J = 7.5, 3.4 Hz, 2H, aromatic), 7.16 (d, J = 8.7 Hz, 2H, aromatic), 6.86 (s, 1H, imidazole), 4.25 (t, J = 7.2 Hz, 2H, CH_A_CH_B_), 3.08 (t, J = 7.2 Hz, 2H, CH_A_CH_B_). ^13^C NMR (125 MHz, DMSO-d_6_) 140.45, 140.33, 139.05, 129.20, 129.12, 128.30, 128.00, 127.66, 127.32, 126.91, 125.06, 119.56, 47.45, 36.95. Anal. Calcd. for (C_17_H_16_N_2_): C, 82.22; H, 6.49; N, 11.28. Found: C, 82.25; H, 6.54; N, 11.33.

### 3.3. General Procedure for the Synthesis of 1-substituted-2-(1H-imidazol-1-yl)ethanones (**4a**–**f**)

The appropriate ketone (5.0 mmol), commercially available or prepared as previously reported [32,33,34], was dissolved in a mixture of EtOAc/CHCl_3_ 1:1 (20 mL). CuBr_2_ (II) was added, and the reaction mixture was left stirring and refluxing for 5 h. After completion, the obtained inorganic material was filtered through a celite pad and washed with EtOAc (20 mL). The filtrate was evaporated, and the residue was crystallized with cyclohexane to obtain the crude 2-bromo-1-substituted ethanones (**3a**–**f**), which were used without further purification in the next step. The appropriate compounds **3a**–**f** were dissolved in anhydrous DMF (15 mL) and added dropwise to a previously prepared suspension of imidazole (15 mmol) and K_2_CO_3_ (15 mmol) in anhydrous DMF (20 mL). The reaction mixture was left stirring for 2 h. Then, water was added, and the resulting suspension was filtered in vacuum. The residue was purified by flash chromatography or by column chromatography using a Biotage^®^ chromatographic system with Biotage^®^ SNAP KP-Sil flash chromatography cartridges using different mixtures of EtOAc and MeOH or DCM and MeOH, to obtain pure **4a**–**f**. By means of this procedure, the following pure compounds were obtained:

#### 3.3.1. 1-[3-[(4-bromobenzyl)oxy]phenyl]2-(1H-imidazol-1-yl)ethanone (**4a**)

Yellow solid (Biotage^®^, 9 EtOAc: 1 MeOH); mp 114.8–118.3 °C; yield 33%. ^1^H NMR (200 MHz, DMSO-*d*_6_): 7.63–7.42 (m, 8H aromatic + 1H imidazole), 7.11 (s, 1H, imidazole), 6.92 (s, 1H, imidazole), 5.72 (s, 2H, CH_2_N), 5.19 (s, 2H, CH_2_O).

#### 3.3.2. 1-[2-[(4-bromobenzyl)oxy]phenyl]-2-(1H-imidazol-1-yl)ethanone (**4b**)

Brown oil (Biotage^®^, 9 EtOAc: 1 MeOH); yield 35%. ^1^H NMR (200 MHz, DMSO-*d*_6_): 7.78 (d, *J* = 9.6 Hz, 1H, imidazole), 7.66–7.53 (m, 6H, aromatic), 7.29 (d, *J* = 8.4 Hz, 1H, aromatic), 7.12 (t, *J* = 7.4 Hz, 1H aromatic + 1H imidazole), 6.88 (s, 1H, imidazole), 5.47 (s, 2H, CH_2_N), 5.32 (s, 2H, CH_2_O).

#### 3.3.3. 1-[4-[(3-bromobenzyl)oxy]phenyl]-2-(1H-imidazol-1-yl)ethanone (**4c**)

Yellow solid (Biotage^®^, 9 EtOAc: 1 MeOH); mp 153.8–160 °C; yield 72%. ^1^H NMR (200 MHz, DMSO-*d*_6_): 8.00 (d, *J* = 8.8 Hz, 2H, aromatic), 7.67 (s, 1H, imidazole), 7.57–7.49 (m, 3H, aromatic), 7.38 (t, *J* = 7 Hz, 1H, aromatic), 7.18 (d, *J* = 8.8 Hz, 1H, imidazole), 7.09 (s, 2H, aromatic), 6.90 (s, 1H, imidazole), 5.66 (s, 2H, CH_2_N), 5.25 (s, 2H, CH_2_O).

#### 3.3.4. 1-[4-[(2-bromobenzyl)oxy]phenyl]-2-(1H-imidazol-1-yl)ethanone (**4d**)

Yellow solid (flash column chromatography, 9.5 DCM: 0.5 MeOH); mp 93.6–100 °C; yield 38%. ^1^H NMR (200 MHz, DMSO-*d*_6_): 8.04 (d, *J* = 8.6 Hz, 2H, aromatic), 7.72 (d, *J* = 7.8 Hz, 1H, imidazole), 7.62 (d*, J* = 8 Hz, 2H, aromatic), 7.49–7.35 (m, 2H, aromatic), 7.22 (d, *J* = 8.8 Hz, 2H, aromatic), 7.11 (s, 1H, imidazole), 6.92 (s, 1H, imidazole), 5.69 (s, 2H, CH_2_N), 5.26 (s, 2H, CH_2_O).

#### 3.3.5. 1-[3-[(3-bromobenzyl)oxy]phenyl]-2-(1H-imidazol-1-yl)ethanone (**4e**)

Brown solid (flash column chromatography, 9.5 EtOAc: 0.5 MeOH); mp 115–117 °C; yield 42%. ^1^H NMR (200 MHz, DMSO-*d*_6_): 7.69 (s, 1H, imidazole), 7.63–7.47 (m, 6H, aromatic), 7.41–7.33 (m, 2H, aromatic), 7.12 (s, 1H, imidazole), 6.92 (s, 1H, imidazole), 5.73 (s, 2H, CH_2_N), 5.22 (s, 2H, CH_2_O).

#### 3.3.6. 1-[3-[(2-bromobenzyl)oxy]phenyl]-2-(1H-imidazol-1-yl)ethanone (**4f**)

Brown solid (flash column chromatography, 9.5 DCM: 0.5 MeOH); mp 105–110 °C; yield 32%. ^1^H NMR (200 MHz, DMSO-*d*_6_): 7.72–7.29 (m, 8H aromatic + 1H imidazole), 7.13 (s, 1H, imidazole), 6.93 (s, 1H, imidazole), 5.74 (s, 2H, CH_2_N), 5.22 (s, 2H, CH_2_O).

### 3.4. General Procedure for the Synthesis of 1-(substituted)-2-(1H-imidazol-1-yl)ethanoles **5a**–**f**

A mixture of the appropriate imidazole-ketone (**4a**–**f**, 0.35 mmol) and NaBH_4_ (0.7 mmol) in anhydrous methanol (10 mL) was refluxed for 2 h. Then, it was evaporated to dryness, added with deionized water (40 mL), acidified with HCl 1 N and heated to 110 °C for 30 min. After cooling to room temperature, the reaction mixture was treated with NaOH 1 N up to a pH 8.5 and the obtained suspension was filtered, washed with water to neutrality, and dried. For some of the final compounds recrystallization with opportune solvent was used to obtained the following pure alcohols:

#### 3.4.1. 1-[3-[(4-bromobenzyl)oxy]phenyl]-2-(1H-imidazol-1-yl)ethanol (**5a**)

Yellow solid (cyclohexane); mp 123–126 °C; yield 70%. ^1^H NMR (500 MHz, DMSO-*d*_6_): 7.59 (d, *J* = 8.4 Hz, 2H, aromatic), 7.49 (s, 1H, imidazole), 7.41 (d, *J* = 8.4 Hz, 2H, aromatic), 7.24 (t, *J* = 7.9 Hz, 1H, aromatic), 7.10 (s, 1H, imidazole), 7.00 (s, 1H, aromatic), 6.94 (d, *J* = 7.6 Hz, 1H, aromatic), 6.89 (d, *J* = 8.2 Hz, 1H, aromatic), 6.83 (s, 1H, imidazole), 5.71 (d, *J* = 4.4 Hz, 1H, OH), 5.06 (s, 2H, CH_2_O), 4.82–4.75 (m, 1H, CH), 4.13 (dd, *J* = 13.9 Hz, *J* = 3.9 Hz, 1H, CH*_A_*CH_B_), 4.02 (dd, *J* = 13.9 Hz, *J* = 7.9 Hz, 1H, CH_A_CH*_B_*). ^13^C NMR (125 MHz, DMSO-*d*_6_) 158.05, 144.39, 136.61, 131.35, 129.73, 129.20, 127.71, 120.89, 120.07, 118.61, 113.61, 112.60, 71.94, 68.33, 53.50. Anal. Calcd. for (C_18_H_17_BrN_2_O_2_): C, 57.92; H, 4.59; N, 7.51. Found: C, 57.95; H, 4.62; N, 7.55.

#### 3.4.2. 1-[2-[(4-bromobenzyl)oxy]phenyl]-2-(1H-imidazol-1-yl)ethanol (**5b**)

Yellow solid (cyclohexane); mp 121.5–130 °C; yield 64%. ^1^H NMR (500 MHz, DMSO-*d*_6_): 7.60 (d, *J* = 8.3 Hz, 2H, aromatic), 7.46 (d, *J* = 8.2 Hz, 3H aromatic + 1H imidazole), 7.35 (d, *J* = 7.1 Hz, 1H, aromatic), 7.27–7.21 (m, 1H, aromatic), 7.05 (d, *J* = 8.2 Hz, 1H, aromatic), 6.97–6.92 (m, 1H, aromatic), 6.89 (s, 1H, imidazole), 6.84 (s, 1H, imidazole), 5.70 (s, 1H, OH), 5.11 (d, *J* = 4.2 Hz, 2H, CH_2_O), 5.06 (d, *J* = 5.6 Hz, 1H, CH), 4.15 (dd, *J* = 13.9 Hz, *J* = 2.7 Hz, 1H, CH*_A_*CH_B_), 3.94 (dd, *J* = 13.9 Hz, *J* = 7.7 Hz, 1H, CH_A_CH*_B_*). ^13^C NMR (125 MHz, DMSO-*d*_6_) 154.60, 136.86, 131.72, 130.49, 130.19, 128.81, 126.79, 121.37, 120.97, 112.02, 68.90, 67.37, 52.69. Anal. Calcd. for (C_18_H_17_BrN_2_O_2_): C, 57.92; H, 4.59; N, 7.51. Found: C, 57.89; H, 4.64; N, 7.53.

#### 3.4.3. 1-[4-[(3-bromobenzyl)oxy]phenyl]-2-(1H-imidazol-1-yl)ethanol (**5c**)

White solid (cyclohexane); mp 120.1–123 °C; yield 84%. ^1^H NMR (500 MHz, DMSO-*d*_6_): 7.64 (s, 1H, imidazole), 7.52 (d, *J* = 7.9 Hz, 1H, aromatic), 7.49–7.42 (m, 2H, aromatic), 7.36 (t, *J* = 7.8 Hz, 1H, aromatic), 7.25 (d, *J* = 8.5 Hz, 2H, aromatic), 7.09 (s, 1H, imidazole), 6.97 (d, *J* = 8.6 Hz, 2H, aromatic), 6.82 (s, 1H, imidazole), 5.60 (d, *J* = 4.4 Hz, 1H, OH), 5.11 (s, 2H, CH_2_O), 4.75 (m, 1H, CH), 4.09 (dd, *J* = 13.9 Hz, *J* = 4.2 Hz, 1H, CH*_A_*CH_B_), 4.01 (dd, *J* = 13.9, 7.8 Hz, 1H, CH_A_CH*_B_*). ^13^C NMR (125 MHz, DMSO-*d*_6_) 157.34, 140.02, 135.10, 130.65, 130.60, 130.12, 127.70, 127.26, 126.52, 121.67, 120.01, 114.41, 71.65, 68.17, 53.56. Anal. Calcd. for (C_18_H_17_BrN_2_O_2_): C, 57.92; H, 4.59; N, 7.51. Found: C, 57.96; H, 4.64; N, 7.56.

#### 3.4.4. 1-[4-[(2-bromobenzyl)oxy]phenyl]-2-(1H-imidazol-1-yl)ethanol (**5d**)

Yellow solid (cyclohexane); mp 120–126 °C; yield 88%. ^1^H NMR (500 MHz, DMSO-*d*_6_): 7.68 (d, *J* = 7.9 Hz, 1H, imidazole), 7.58 (d, *J* = 7.3 Hz, 1H, aromatic), 7.49 (s, 1H, aromatic), 7.43 (t, *J* = 7.4 Hz, 1H, aromatic), 7.34–7.25 (m, 3H, aromatic), 7.11 (s, 1H, imidazole), 6.98 (d, *J* = 8.6 Hz, 2H, aromatic), 6.83 (s, 1H, imidazole), 5.62 (d, *J* = 4.5 Hz, 1H, OH), 5.11 (s, 2H, CH_2_O), 4.76 (m, 1H, CH), 4.10 (dd, *J* = 13.8, 4.1 Hz, 1H, CH*_A_*CH_B_), 4.01 (dd, *J* = 13.9, 8.0 Hz, 1H, CH_A_CH*_B_*). ^13^C NMR (125 MHz, DMSO-*d*_6_) 157.44, 135.95, 135.27, 132.66, 130.25, 127.93, 127.65, 127.34, 122.84, 120.04, 114.33, 71.69, 69.07, 53.58. Anal. Calcd. for (C_18_H_17_BrN_2_O_2_): C, 57.92; H, 4.59; N, 7.51. Found: C, 57.94; H, 4.63; N, 7.49.

#### 3.4.5. 1-[3-[(3-bromobenzyl)oxy]phenyl]-2-(1H-imidazol-1-yl)ethanol (**5e**)

White solid (diethyl ether); mp 93.5–99.4 °C; yield 79%. ^1^H NMR (500 MHz, DMSO-*d*_6_): 7.66 (s, 1H, imidazole), 7.57–7.43 (m, 3H, aromatic), 7.37 (t, *J* = 7.8 Hz, 1H, aromatic), 7.25 (t, *J* = 7.9 Hz, 1H, aromatic), 7.11 (s, 1H, imidazole), 7.02 (s, 1H, aromatic), 6.97–6.88 (m, 2H, aromatic), 6.84 (s, 1H, aromatic), 5.72 (s, 1H, OH), 5.10 (s, 2H, CH_2_O), 4.84–4.73 (m, 1H, CH), 4.13 (dd, *J* = 13.9, 3.8 Hz, 1H, CH*_A_*CH_B_), 4.02 (dd, *J* = 13.9, 8.0 Hz, 1H, CH_A_CH*_B_*). ^13^C NMR (125 MHz, DMSO-*d*_6_) 158.00, 144.47, 140.00, 130.70, 130.65, 130.16, 129.24, 127.76, 126.56, 121.72, 120.09, 118.67, 113.55, 112.60, 71.96, 68.12, 53.53. Anal. Calcd. for (C_18_H_17_BrN_2_O_2_): C, 57.92; H, 4.59; N, 7.51. Found: C, 57.96; H, 4.57; N, 7.53.

#### 3.4.6. 1-[3-[(2-bromobenzyl)oxy]phenyl]-2-(1H-imidazol-1-yl)ethanol (**5f**)

Yellow solid (cyclohexane); mp 132–140 °C; yield 86%. ^1^H NMR (500 MHz, DMSO-*d*_6_): 7.68 (d, *J* = 8.0 Hz, 1H, imidazole), 7.56 (t, *J* = 11.3 Hz, 2H, aromatic), 7.43 (t, *J* = 7.5 Hz, 1H, aromatic), 7.29 (m, 2H, aromatic), 7.14 (s, 1H, aromatic), 7.01 (s, 1H, imidazole), 6.96 (d, *J* = 7.6 Hz, 1H, aromatic), 6.91 (d, *J* = 8.1 Hz, 1H, aromatic), 6.86 (s, 1H, aromatic), 5.77 (s, 1H, OH), 5.08 (s, 2H, CH_2_O), 4.80 (d, *J* = 3.8 Hz, 1H, CH), 4.15 (dd, *J* = 13.9, 3.8 Hz, 1H), 4.03 (dd, *J* = 13.9, 8.0 Hz, 1H). ^13^C NMR (125 MHz, DMSO-*d*_6_) 158.20, 144.54, 136.03, 132.80, 130.41, 130.31, 129.47, 128.10, 122.99, 118.91, 113.73, 112.53, 72.03, 69.13, 53.67. Anal. Calcd. for (C_18_H_17_BrN_2_O_2_): C, 57.92; H, 4.59; N, 7.51. Found: C, 57.94; H, 4.61; N, 7.48.

### 3.5. General Procedure for the Synthesis of Benzyl Derivatives (**6a**,**b**)

Compound A (0.56 mmol) was dissolved in dry DMF (3 mL) and NaH 80% dispersion in mineral oil (0.70 mmol) was added. The suspension was added dropwise with a solution of the appropriate benzyl bromide (0.67 mmol) previously dissolved in 1.5 mL of dry DMF. The mixture was left stirring for 2 h, added with 5 mL of methanol and concentrated to reduced volume under vacuum. The residue was diluted with water (50 mL) and NaOH 1N (1 mL), and extracted with EtOAc (3 × 70 mL). The combined organic layers were washed with brine, dried over anhydrous Na_2_SO_4_, filtered, and evaporated. The obtained crude material was purified by column chromatography using EtOAc (100%) as eluent, to afford the title compound as pure oil.

#### 3.5.1. 1-(2-(3-bromophenyl)-2-((4-chlorobenzyl)oxy)ethyl)-1H-imidazole (**6a**)

Colorless oil; yield 33%. ^1^H NMR (500 MHz, DMSO-*d*_6_): 7.56 (d, *J* = 11.5 Hz, 2H aromatic + 1H imidazole), 7.36 (m, 4H, aromatic), 7.17–7.11 (m, 3H, aromatic), 6.88 (s, 1H, imidazole), 4.76–4.69 (m, 1H, CH), 4.38 (d, *J* = 12.5 Hz, 1H, CH), 4.27 (s, 1H, CH), 4.24 (d, *J* = 5.0 Hz, 1H, CH), 4.03 (dd, *J* = 14.2, 7.1 Hz, 1H, CH). ^13^C NMR (125 MHz, DMSO-*d*_6_) 141.58, 136.96, 132.03, 131.12, 130.84, 129.66, 128.96, 128.28, 128.04, 125.79, 121.94, 120.14, 79.54, 69.40, 51.59. Anal. Calcd. for (C_18_H_16_BrClN_2_O): C, 55.19; H, 4.12; N, 7.15. Found: C, 55.17; H, 4.10; N, 7.19.

#### 3.5.2. 1-(2-(3-bromophenyl)-2-((2,5-dichlorobenzyl)oxy)ethyl)-1H-imidazole (**6b**)

Yellow oil; yield 65%. ^1^H NMR (500 MHz, DMSO-*d*_6_): 7.61 (s, 1H, imidazole), 7.55 (d, *J* = 11.3 Hz, 2H, aromatic), 7.45–7.33 (m, 4H, aromatic), 7.31 (d, *J* = 2.5 Hz, 1H, aromatic), 7.15 (s, 1H, imidazole), 6.87 (s, 1H, imidazole), 4.85 (dd, *J* = 8.0, 4.0 Hz, 1H, CH), 4.44–4.35 (m, 2H, CH_2_O), 4.28 (m, 2H, CH_2_N). ^13^C NMR (125 MHz, DMSO-*d*_6_) 141.34, 137.83, 137.49, 131.95, 131.18, 130.75, 130.71, 130.29, 129.68, 128.99, 128.48, 128.10, 125.79, 121.87, 119.91, 80.11, 67.10, 51.43. Anal. (C_18_H_15_BrCl_2_N_2_O) C, H, N, O. Anal. Calcd. for (C_18_H_15_BrCl_2_N_2_O): C, 50.73; H, 3.55; N, 6.57. Found: C, 50.77; H, 3.59; N, 6.53.

### 3.6. Biological Evaluation

#### 3.6.1. Preparation of Spleen and Brain Microsomal Fractions

The dominance of HO-1 protein in the rat spleen and of HO-2 in the rat brain has been well documented [40]; thus, the microsomal fraction, prepared by differential centrifugation, from rat spleen and brain have been used to obtain HO-1 and HO-2. These particular microsomal preparations were selected in order to use the most native (i.e., closest to in vivo) forms of HO-1 and HO-2. Spleen and brain microsomal fractions, from Sprague–Dawley rats, were prepared following a published procedure [41]. All experiments complied with current Italian law and met the guidelines of the Institutional Animal Care and Use Committee of MINISTRY OF HEALTH (Directorate General for Animal Health and Veterinary Medicines, Italy). Male Sprague–Dawley albino rats (150 g body weight and age 45 d) were used. Rats had free access to water, and they were maintained at room temperature with a natural photoperiod (12 h:12 h light–dark cycle). For measuring HO-1 and HO-2 activities, each rat was sacrificed and their spleen and brain were excised and weighed. A homogenate (15%, *w*/*v*) of spleens and brains pooled from four rats was prepared in ice-cold HO-homogenizing buffer (50 mM Tris buffer, pH 7.4, containing 0.25 M sucrose) using a Potter–Elvehjem homogenizing system with a Teflon pestle. The microsomal fraction of rat spleen and brain homogenate was obtained by centrifugation at 10,000× *g* for 20 min at 4 °C, followed by centrifugation of the supernatant at 100,000× *g* for 60 min at 4 °C. The 100,000× *g* pellet (microsomes) was resuspended in 100 mM potassium phosphate buffer, pH 7.8, containing 2 mM MgCl_2_ with a Potter–Elvehjem homogenizing system. Equal aliquots of the rat spleen and brain microsomal fractions were prepared, placed into microcentrifuge tubes, and stored at −80 °C for up to 2 months. Finally, the Lowry method was used to determine the protein concentration of the microsomal fraction [42].

#### 3.6.2. Preparation of Biliverdin Reductase

Liver cytosol has been used as a source of biliverdin reductase. Rat liver was perfused through the hepatic portal vein with cold 0.9% NaCl, then it was cut and flushed with 2 × 20 mL of ice-cold PBS to remove all of the blood. Liver tissue was homogenized in three volumes of a solution containing 1.15% KCl *w*/*v* and Tris buffer 20 mM, pH 7.8 on ice. Homogenates were centrifuged at 10,000× *g* for 20 min at 4 °C. The supernatant was decanted and centrifuged at 100,000× *g* for 1 h at 4 °C to sediment the microsomes. The 100,000× *g* supernatant was saved and then stored in small amounts at −80 °C after its protein concentration was measured. 

#### 3.6.3. Measurement of HO-1 and HO-2 Enzymatic Activities in the Microsomal Fraction of Rat Spleen and Brain

The measurement of bilirubin formation, using the difference in absorbance at 464–530 nm, was used to determine the HO-1 and HO-2 activities, as previously described by Ryter et al. [41]. Reaction mixtures (500 μL) consisted of 20 mM Tris–HCl, pH 7.4, (1 mg/mL) microsomal extract, 0.5–2 mg/mL biliverdin reductase, 1 mM NADPH, 2 mM glucose 6-phosphate (G6P), 1 U G6P dehydrogenase, 25 μM hemin, and 10 μL of DMSO (or the same volume of DMSO solution of test or reference compounds to a final concentration of 100, 10, and 1 μM). Incubations were carried out for 60 min at 37 °C in a circulating water bath in the dark. One volume of chloroform was added to stop the reactions. Then, the chloroform phase was recovered, and the amount of bilirubin, which was formed has been measured with a double-beam spectrophotometer as OD464–530 nm (extinction coefficient, 40 mM/cm^−1^ for bilirubin). The amount of enzyme catalyzing the formation of 1 nmol of bilirubin/mg protein/h was defined as one unit of the enzyme.

#### 3.6.4. Cell Cultures

MCF-7 and MDA-MB-231 cells were maintained in complete growth media (DMEM/Ham’s F12 supplemented with 10% fetal bovine serum, 2 mM l-glutamine, 100  units/mL penicillin, 100 units/mL of streptomycin, and 2.2 g/L of NaHCO_3_) at 37 °C in a humidified atmosphere of 5% CO_2_. For all procedures, cells were harvested using TrypLE Express (Life Technologies, Auckland, New Zealand).

#### 3.6.5. In Vitro Cytotoxic Activity

Cells were seeded into 96-well plates and incubated for 24 h to test the cytotoxic effect of the HO inhibitor. After 24 h incubation, different concentrations of the HO inhibitor were administered for 48 h. Following the incubation, cells were fixed using 10% trichloro-acetic acid (TCA). The cytotoxicity was determined using the sulforhodamine B assay was used, as previously described [43], to determine the cytotoxicity of the HO-1 inhibitor. IC_50_ (the concentration required to decrease the cell number by 50%) was determined by non-linear regression using Graphpad prism 6 software. Treatments were performed in triplicate, and data represent the mean of three independent experiments.

### 3.7. Computational Methods

The Marvin Sketch (18.24, ChemAxon Ltd., Budapest, Hungary) software was used to generate the 3D structures of the studied compounds [44]. First of all, the Merck molecular force field (MMFF94) present in Marvin Sketch at neutral pH was used to minimize the geometry. Subsequently, the geometry was further optimized using the PM3 Hamiltonian in MOPAC (MOPAC2016 v. 18.151, Stewart Computational Chemistry, Colorado Springs, CO, USA) [45,46]. Following our published procedure, the docking model was validated using a set of well-known HO-1 inhibitors (Azalanstat, QC-15, QC-80, QC-82, QC-86, and QC-308) [32]. For molecules with a chiral center only on the (*S*)-enantiomer was studied as already reported [32]. The protein and the ligands were prepared using YASARA (v. 19.5.5, YASARA Biosciences GmbH, Vienna, Austria). The protein structure was downloaded from the protein data bank (https://www.rcsb.org/, ID: 2DY5), and only chain A and the heme group were maintained. Since water molecules are not involved in complex stabilization, we removed all of them, and they were not considered in the docking process. During the docking experiments, amino acidic residues were kept rigid, while the single bonds of the ligands were free to rotate. The point charges were firstly calculated using the AMBER14 force field and then damped to mimic the less polar Gasteiger charges used to optimize the AutoDock scoring function. Docking was performed by applying the Lamarckian genetic algorithm (LGA) implemented in AutoDock using YASARA GUI. The ligand-centered maps were generated by the AutoGrid with a spacing of 0.375 Å and dimensions that surround all atoms extending 5 Å from the surface of the ligand in a cuboid box. All of the parameters were at their default settings.

## 4. Conclusions

In this work, we designed and synthesized a novel series of imidazole-based HO-1 inhibitors structurally linked to our previously reported arylethanolimidazoles [32]. Being the imidazole ring not easily replaceable without drastic loss of activity, the other two portions of the molecule, i.e., the central spacer and the hydrophobic moiety, were modified. Results of SAR studies indicate that the presence of a hydroxyl group in the central spacer is crucial for optimal HO-1 inhibition. On the contrary, the hydrophobic moiety may be modified, but with some restrictions. In fact, when a 4-bromobenzyloxy substituent is located at the *para* or *meta* position of the central phenyl ring, such as in compounds **C** and **5a**, very good results were obtained, whereas a dramatic reduction of activity was observed when the same substituent was moved at the *orto* position of the same phenyl ring, such as in compound **5b**. Molecular modeling studies helped in understanding the binding mode of these compounds, highlighting that the molecules interact correctly with the HO-1 binding site. Finally, the most potent and selective analog (**5a**) showed a moderate cytotoxic effect (IC_50_ = 47.36 ± 6.8 µM) towards MCF-7 breast cancer cells in the SRB assay. The evaluation of HO-1 inhibitors in other cancer cell lines in which HO-1 is overexpressed, as well as their combination with chemotherapeutic drugs, is currently due to course.

## Figures and Tables

**Table 1:**
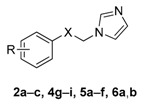
Inhibitory potency of compounds **2a**–**c**, **4g**–**i**, **5a**–**f**, and **6a**, **b** towards HO-1 and HO-2.

Compound	R	X	IC_50_ (μM) ± SD ^a^	
			HO-1	HO-2
**2a**	3-Br	CH_2_	100 ± 5.60	NT
**2b**	4-(4-BrC_6_H_4_)CH_2_O	CH_2_	62.87 ± 3.20	NT
**2c**	3-Ph	CH_2_	46.77 ± 1.80	NT
**4g** ^b^	3-Br	CO	38.17 ± 1.80	NT
**4h** ^b^	3-Ph	CO	19.87 ± 2.50	NT
**4i** ^b^	4-(4-BrC_6_H_4_)CH_2_O	CO	55.46 ± 0.05	NT
**5a**	3-(4-BrC_6_H_4_)CH_2_O	CHOH	0.90 ± 0.02	53.59 ±1.20
**5b**	2-(4-BrC_6_H_4_)CH_2_O	CHOH	>100	NT
**5c**	4-(3-BrC_6_H_4_)CH_2_O	CHOH	41 ± 1.50	NT
**5d**	4-(2-BrC_6_H_4_)CH_2_O	CHOH	9 ± 2.20	15.85 ±1.60
**5e**	3-(3-BrC_6_H_4_)CH_2_O	CHOH	46 ± 1.90	NT
**5f**	3-(2-BrC_6_H_4_)CH_2_O	CHOH	44 ± 1.80	NT
**6a**	3-Br	CHOCH_2_(4-ClC_6_H_4_)	>100	NT
**6b**	3-Br	CHOCH_2_(2,5-ClC_6_H_3_)	66 ± 3.20	NT
**A**	3-Br	CHOH	0.40 ± 0.01 ^c^	32 ± 2.2 ^c^
**B**	3-Ph	CHOH	0.90 ± 0.08 ^c^	>100 ^c^
**C**	4-(4-BrC_6_H_4_)CH_2_O	CHOH	0.95 ± 0.02 ^c^	>100 ^c^
SnPP	-	-	0.58 ± 0.03	0.36 ± 0.01

^a^ Each value is the mean ± SD of three determinations. ^b^ Described in ref [32]**.** ^c^ Data taken from ref [32]**.**

**Table 2:** Docking results for the studied molecules **2a**–**c**, **5a**–**f,** and **6a**, **b**.

Compound	Δ*G*_B_ calcd. (kcal/mol)	*K*_i_ calcd. (μM)	IC_50_ exp. (μM) HO-1
**2a**	−5.71	64.92	100
**2b**	−5.91	46.31	62.87 ± 3.20
**2c**	−6.10	33.60	46.77 ± 1.80
**5a**	−8.42	0.66	0.9 ± 0.02
**5b**	−5.48	95.74	>100
**5c**	−5.38	113.35	41 ± 1.50
**5d**	−6.60	14.44	9 ± 2.20
**5e**	−6.15	30.88	46 ± 1.90
**5f**	−6.15	30.88	44 ± 1.80
**6a**	−4.24	777.11	>100
**6b**	−5.81	54.83	66 ± 3.20

## Supplementary Materials

The following are available online at https://www.mdpi.com/1422-0067/21/6/1923/s1, Table S1: Elemental analysis data for compounds **2a**–**c**, **5a**–**f**, and **6a**,**b**; Figures S1–S22: ^1^H NMR and ^13^C NMR spectra of compounds **2a**–**c**, **5a**–**f**, and **6a**,**b**; Figures S23–S25: Binding mode of compounds **2a**–**c**, **5a**–**f**, and **6a**,**b**; Figures S26–S36: 2D interactions of compounds **2a**–**c**, **5a**–**f**, and **6a**,**b**.
