# Supplementary material for: New Arylethanolimidazole Derivatives as HO-1 Inhibitors with Cytotoxicity against MCF-7 Breast Cancer Cells
Source: Int J Mol Sci. 2020 Mar 11;21(6):1923. doi: 10.3390/ijms21061923 (PMC7139504; doi:10.3390/ijms21061923)
Supplement: Supplementary file 1 [file ijms-21-01923-s001.pdf]

International Journal of *Molecular Sciences*

Supplementary Material

New arylethanolimidazole derivatives as HO-1 inhibitors with cytotoxicity against MCF-7 breast cancer cells

Valeria Ciaffaglione [1], Sebastiano Intagliata [1,*], Valeria Pittalà [1], Agostino Marrazzo [1], Valeria Sorrenti [1], Luca Vanella [1] Antonio Rescifina [1,2], Giuseppe Floresta [1,3], Ameera Sultan [4], Khaled Greish [4], and Loredana Salerno [1,*]

1 Department of Drug Sciences, University of Catania, viale A. Doria 6, 95125, Catania, Italy; valeria.ciaffaglione@phd.unict.it (V.C.); s.intagliata@unict.it (S.I.); vpittala@unict.it (V.P.); marrazzo@unict.it (A.M.); sorrenti@unict.it (V.S.); lvanella@unict.it (L.V.); arescifina@unict.it (A.R.); giuseppe.floresta@unict.it (G.F.); l.salerno@unict.it (L.S.)
2 Consorzio Interuniversitario Nazionale di ricerca in Metodologie e Processi Innovativi di Sintesi (C.I.N.M.P.S.), Via E. Orabona, 4, 70125 Bari, Italy
3 Institute of Pharmaceutical Science, King's College London, Stamford Street, London SE1 9NH, UK
4 Department of Molecular Medicine, College of Medicine and Medical Sciences, Princess Al-Jawhara Centre for Molecular Medicine, Arabian Gulf University, Manama, Kingdom of Bahrain; ameeraa@agu.edu.bh (A.S.); khaledfg@agu.edu.bh (K.G.)

* Correspondence: s.intagliata@unict.it (S.I.); l.salerno@unict.it (L.S.); Tel.: +39-095-738-4053 (S.I.); +39-095-738-4266 (L.S.)

Table of contents

**Table S1**: Elemental analysis data for compounds **2a–c**, **5a–f**, and **6a**,**b**.	S2
**Figures S1–S22**: ^{1}H NMR and ^{13}C NMR spectra of compounds **2a–c**, **5a–f**, and **6a**,**b**.	S3–S13
**Figures S23–S25:** Binding mode of compounds **2a–c**, **5a–f**, and **6a**,**b**.	S14–S15
**Figures S26–S36:** 2D interactions of compounds **2a–c**, **5a–f**, and **6a**,**b**.	S15–S20

Table S1. Elemental analysis data for compounds **2a–c**, **5a–f**, and **6a**,**b**.

			Calcd			Found		
Compd	Formula	Mw	C	H	N	C	H	N
**2a**	$C_{11}H_{11}BrN_2$	251.12	52.61	4.42	11.16	52.69	4.48	11.19
**2b**	$C_{18}H_{17}BrN_2O_2$	357.24	60.52	4.80	7.84	60.48	4.86	7.80
**2c**	$C_{17}H_{16}N_2$	248.32	82.22	6.49	11.28	82.25	6.54	11.33
**5a**	$C_{18}H_{17}BrN_2O_2$	373.24	57.92	4.59	7.51	57.95	4.62	7.55
**5b**	$C_{18}H_{17}BrN_2O_2$	373.24	57.92	4.59	7.51	57.89	4.64	7.53
**5c**	$C_{18}H_{17}BrN_2O_2$	373.24	57.92	4.59	7.51	57.96	4.64	7.56
**5d**	$C_{18}H_{17}BrN_2O_2$	373.24	57.92	4.59	7.51	57.94	4.63	7.49
**5e**	$C_{18}H_{17}BrN_2O_2$	373.24	57.92	4.59	7.51	57.96	4.57	7.53
**5f**	$C_{18}H_{17}BrN_2O_2$	373.24	57.92	4.59	7.51	57.94	4.61	7.48
**6a**	$C_{18}H_{16}BrClN_2O$	391.69	55.19	4.12	7.15	55.17	4.10	7.19
**6b**	$C_{18}H_{15}BrCl_2N_2O$	426.13	50.73	3.55	6.57	50.77	3.59	6.53

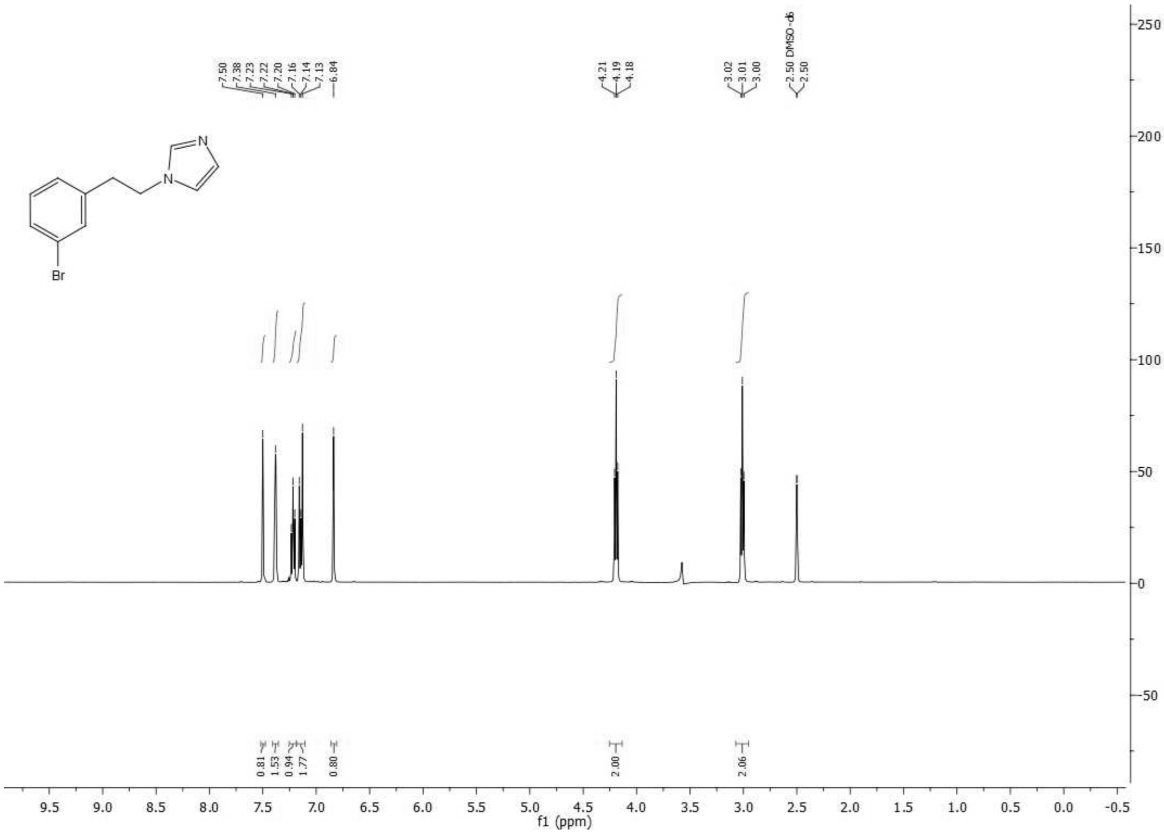

Figure S1. ^{1}H NMR of compound **2a.**

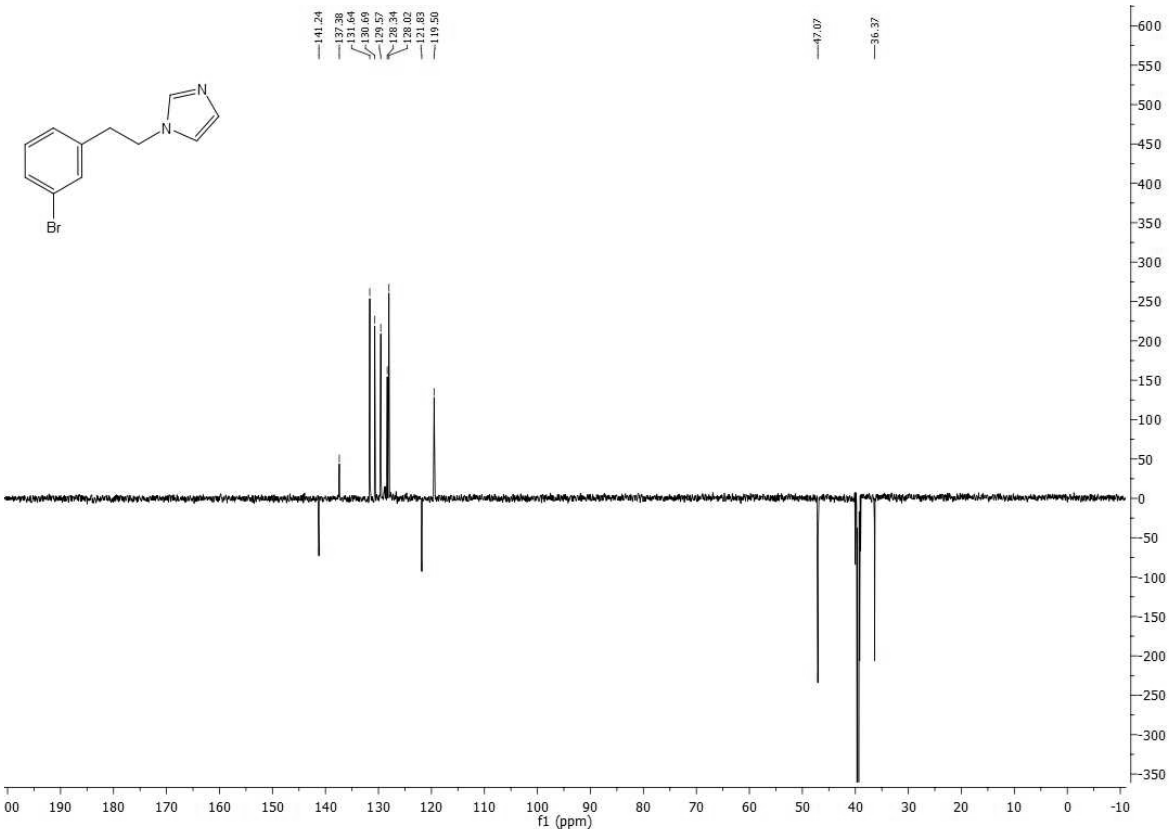

Figure S2. ^{13}C NMR of compound **2a.**

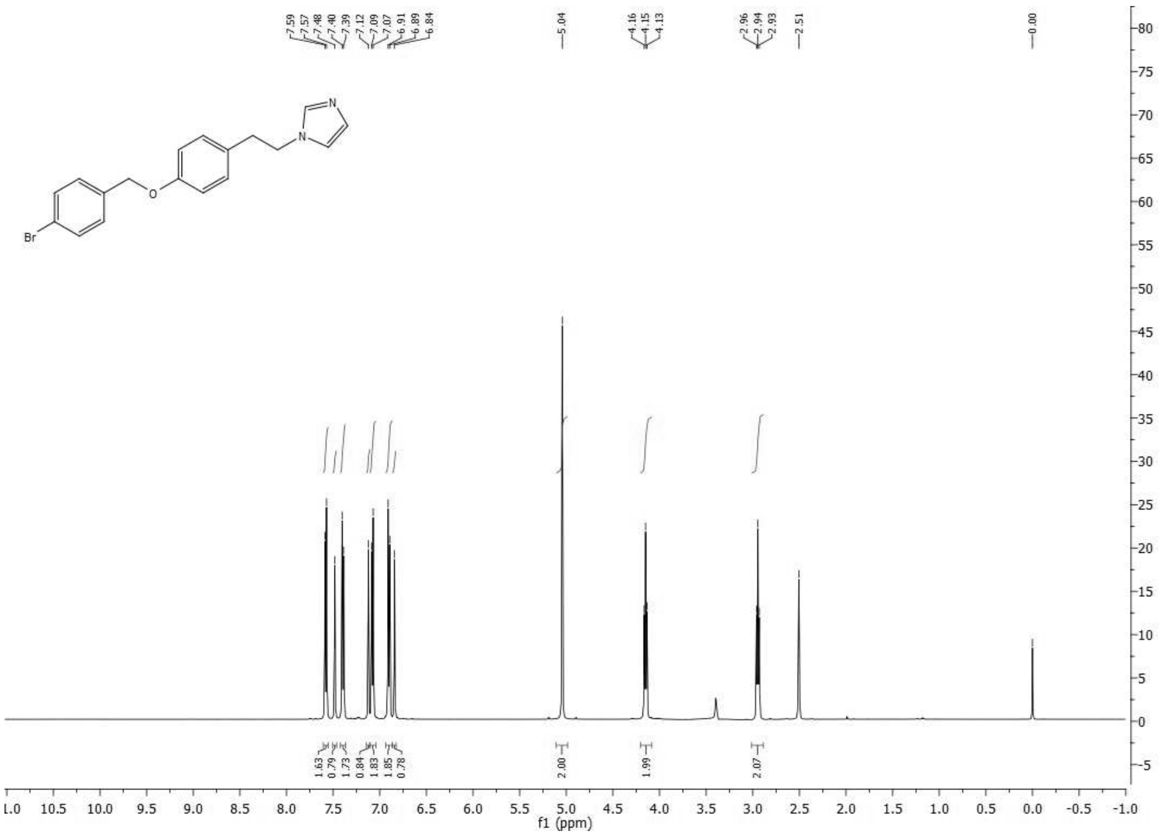

Figure S3. ^{1}H NMR of compound **2b.**

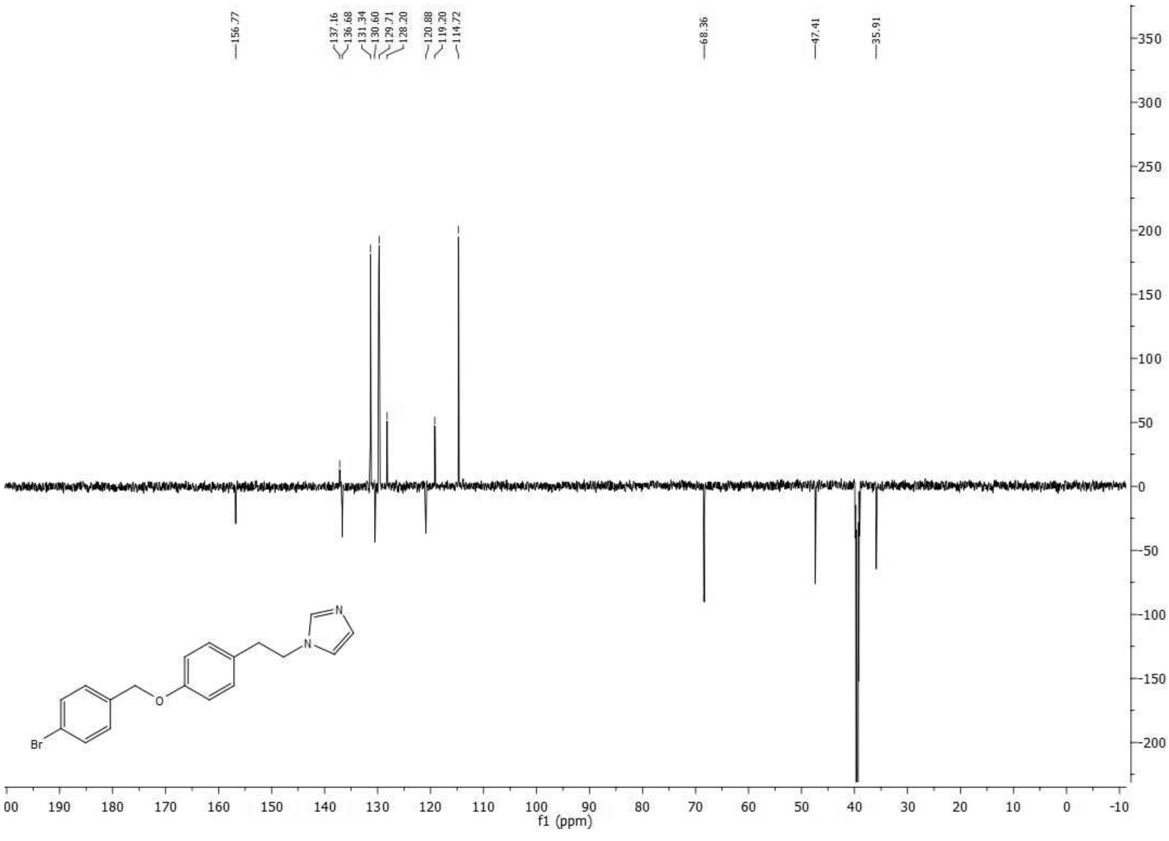

Figure S4. ^{13}C NMR of compound **2b.**

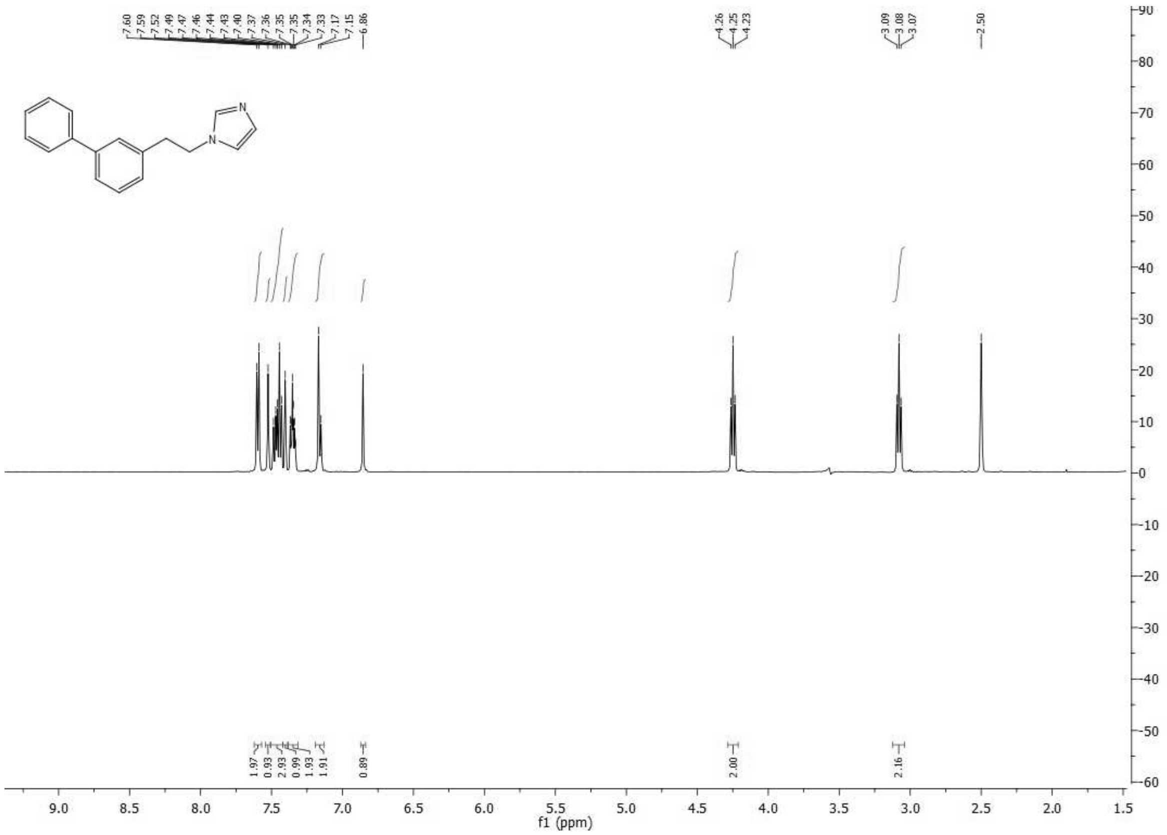

Figure S5. ^{1}H NMR of compound **2c.**

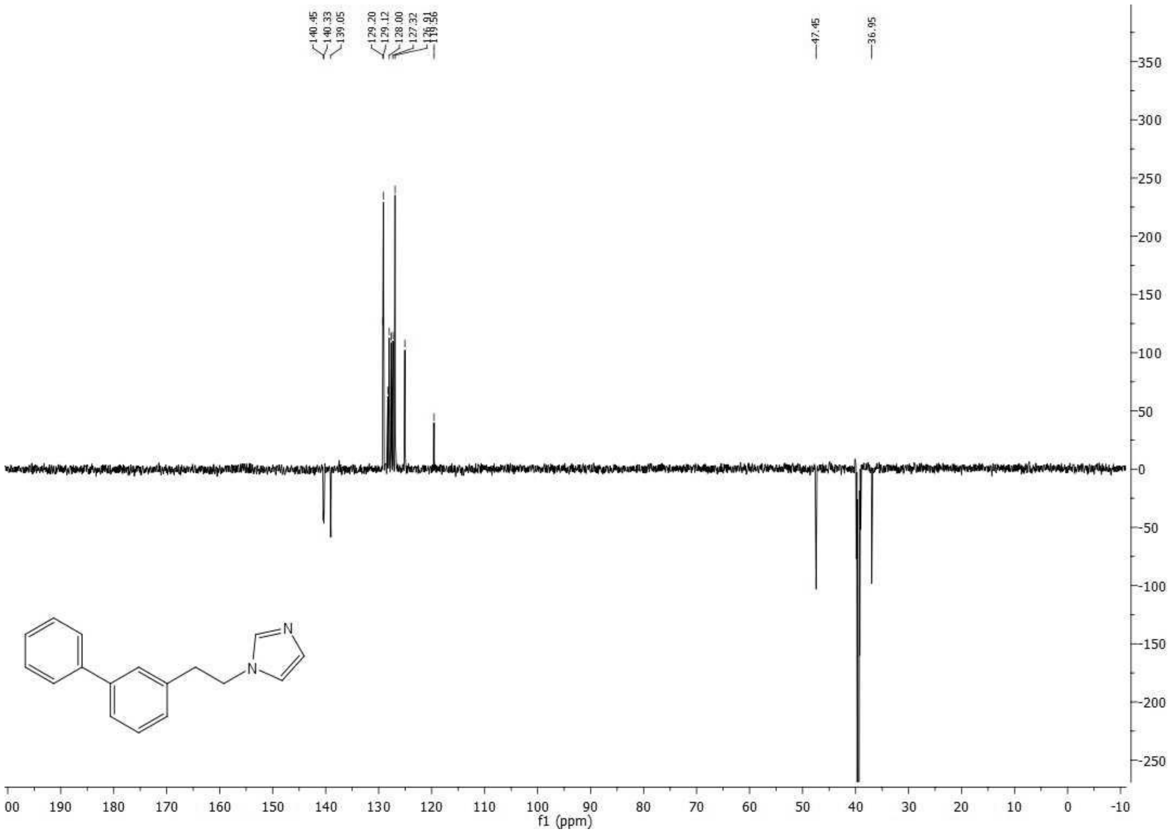

Figure S6. ^{13}C NMR of compound **2c.**

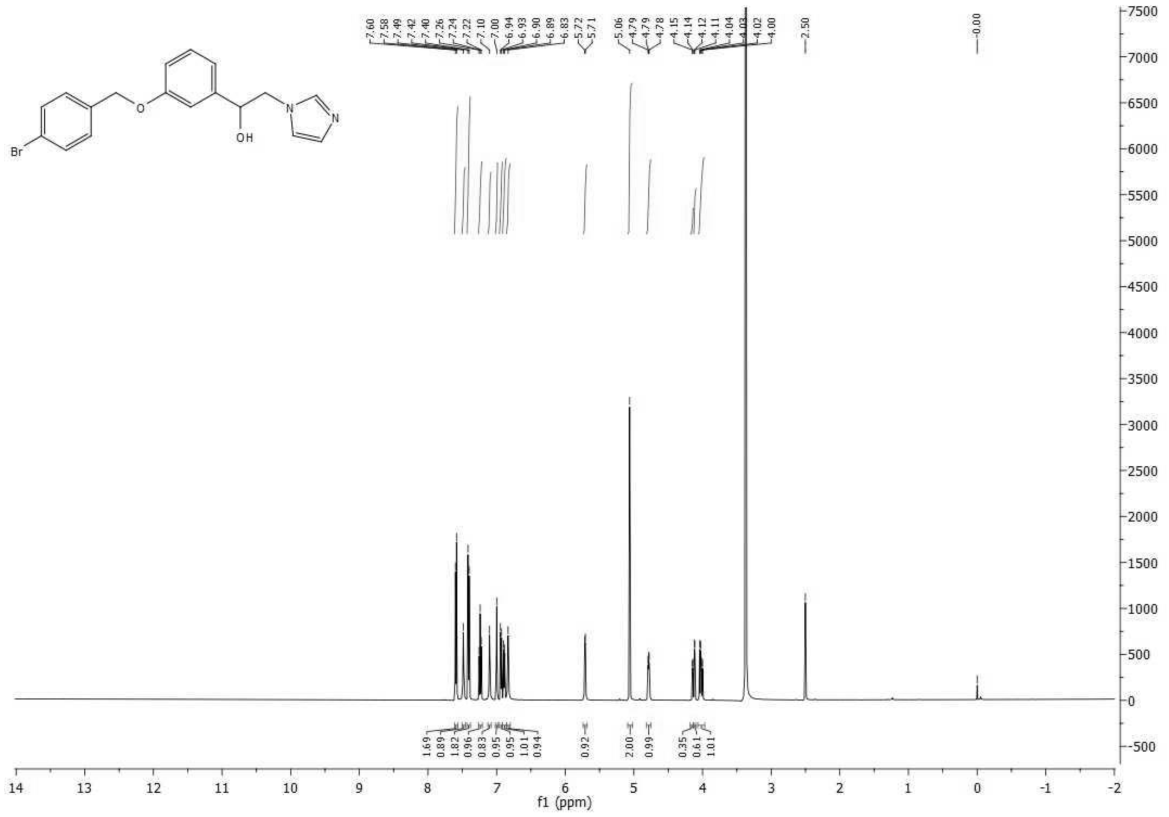

Figure S7. ^{1}H NMR of compound **5a.**

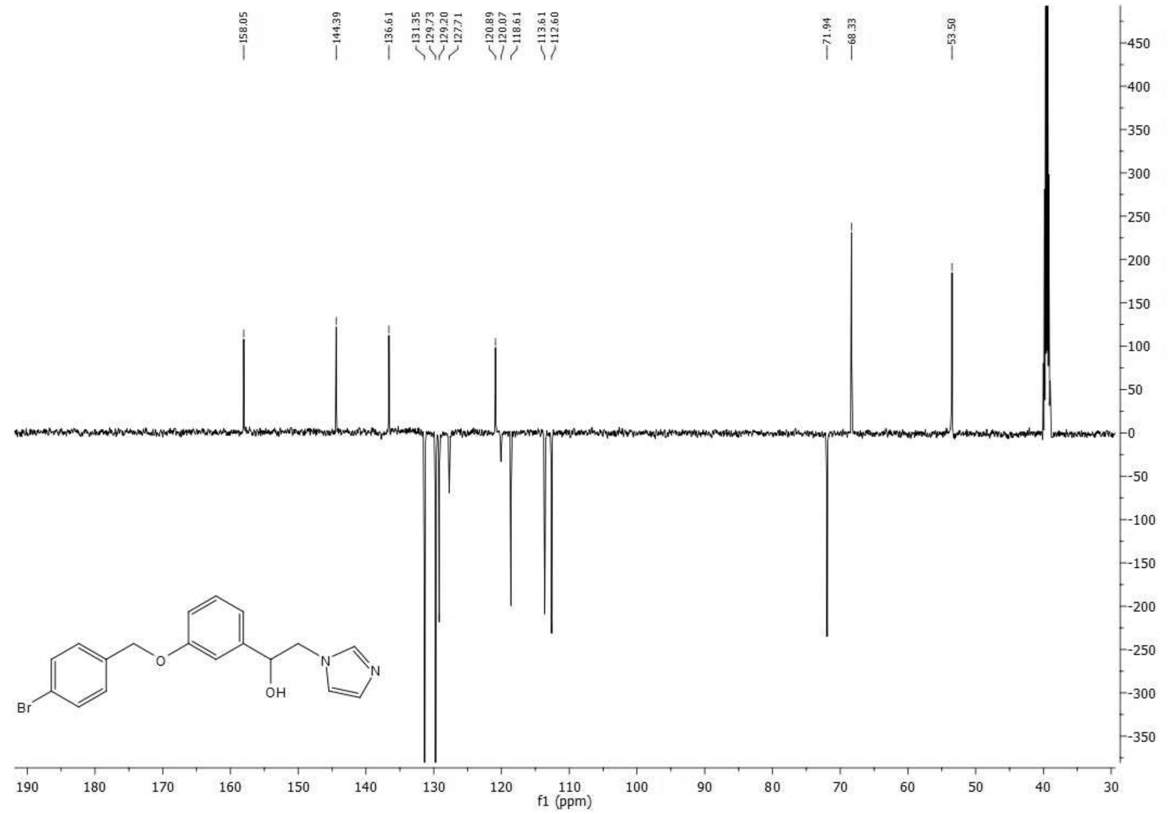

Figure S8. ^{13}C NMR of compound **5a.**

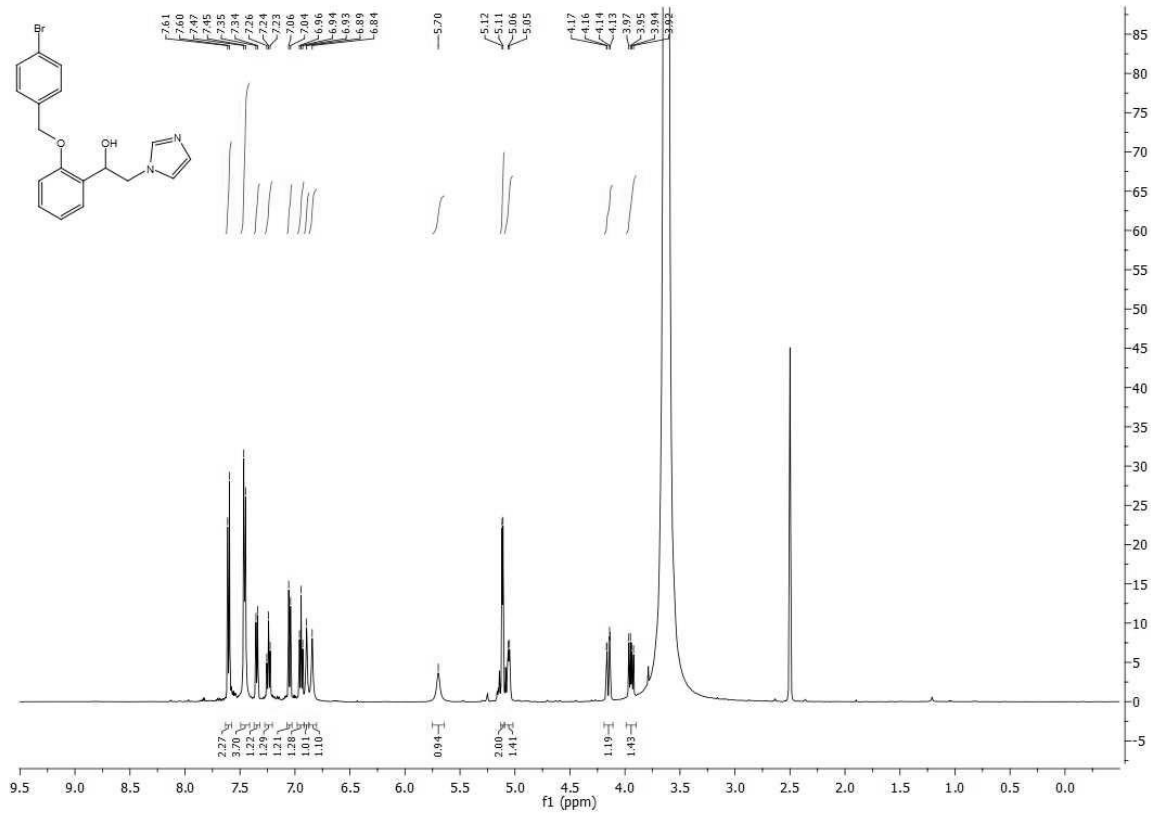

Figure S9. ^{1}H NMR of compound **5b.**

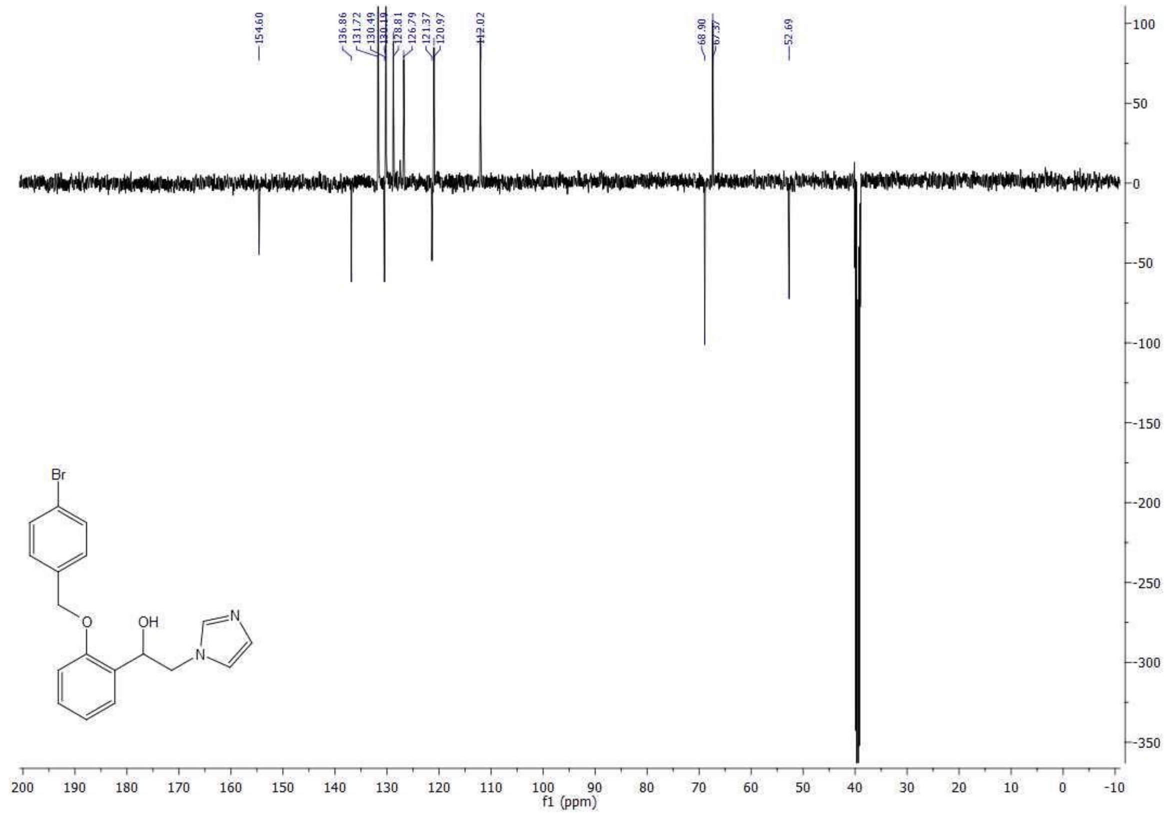

Figure S10. ^{13}C NMR of compound **5b.**

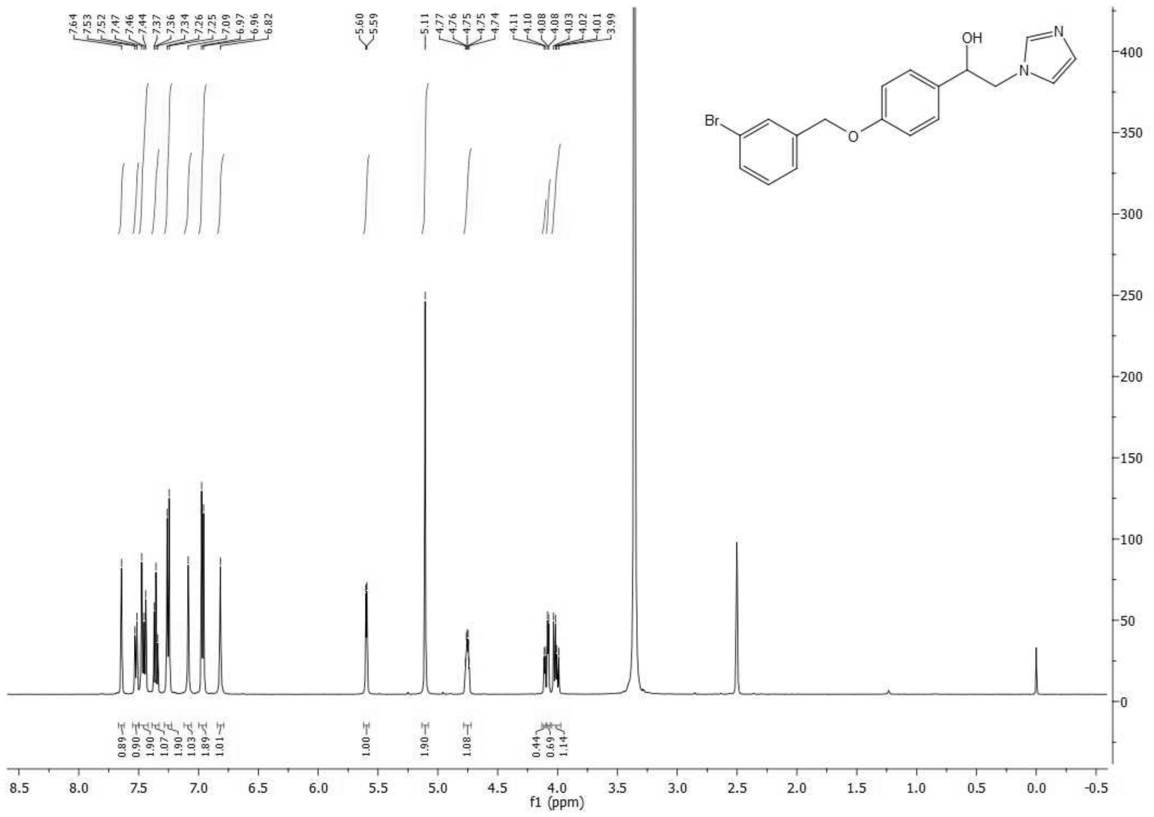

Figure S11. ^{1}H NMR of compound **5c.**

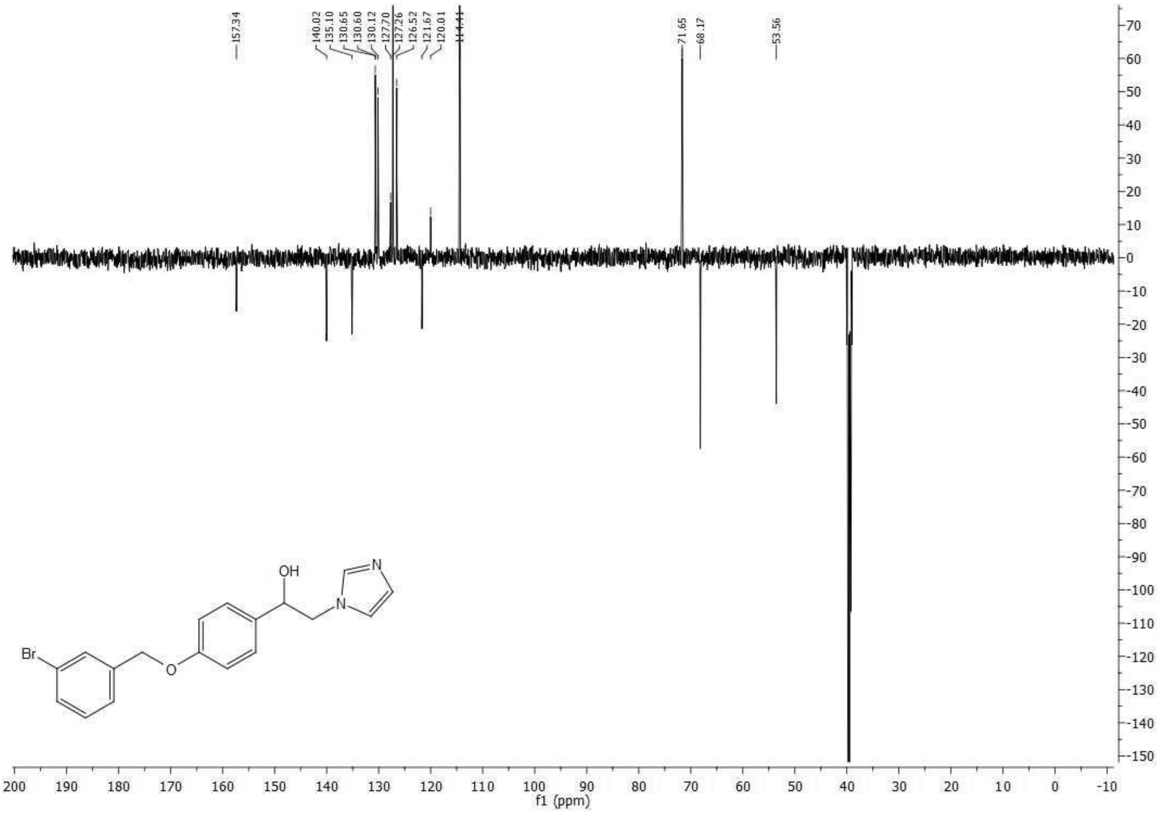

Figure S12. ^{13}C NMR of compound **5c.**

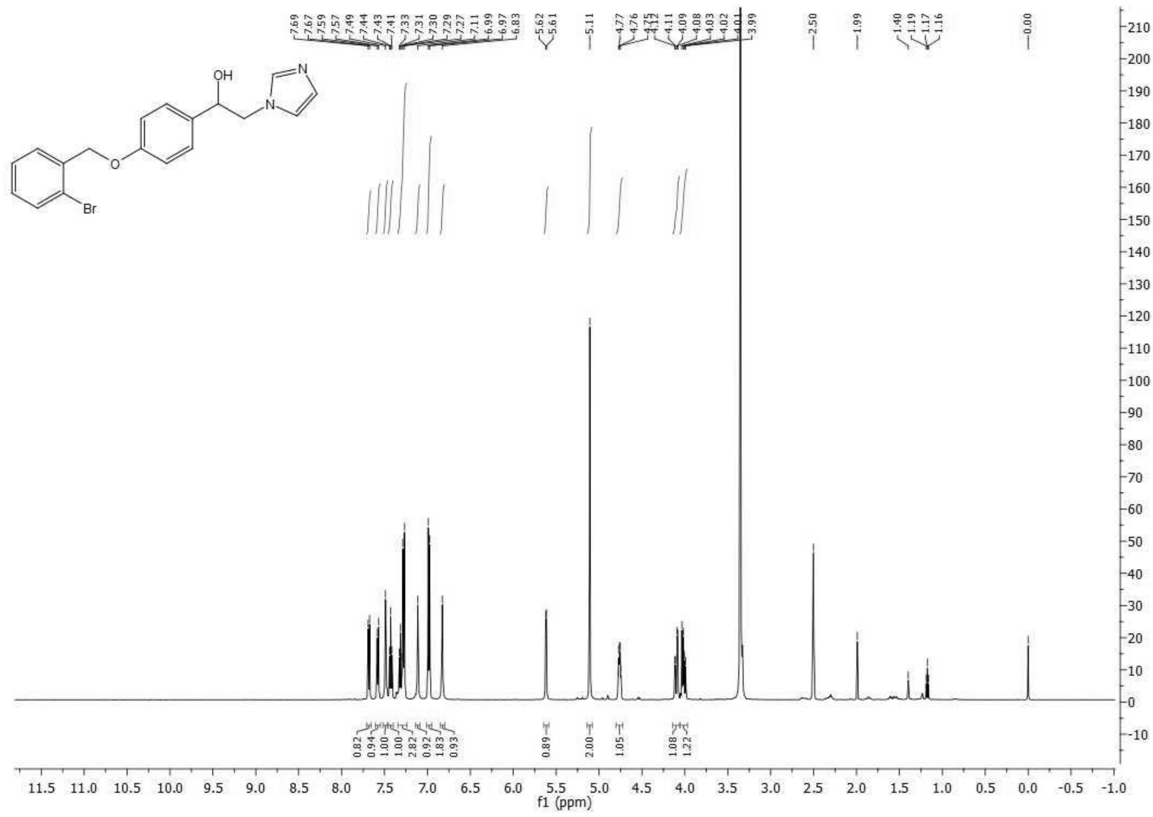

Figure S13. ^{1}H NMR of compound **5d.**

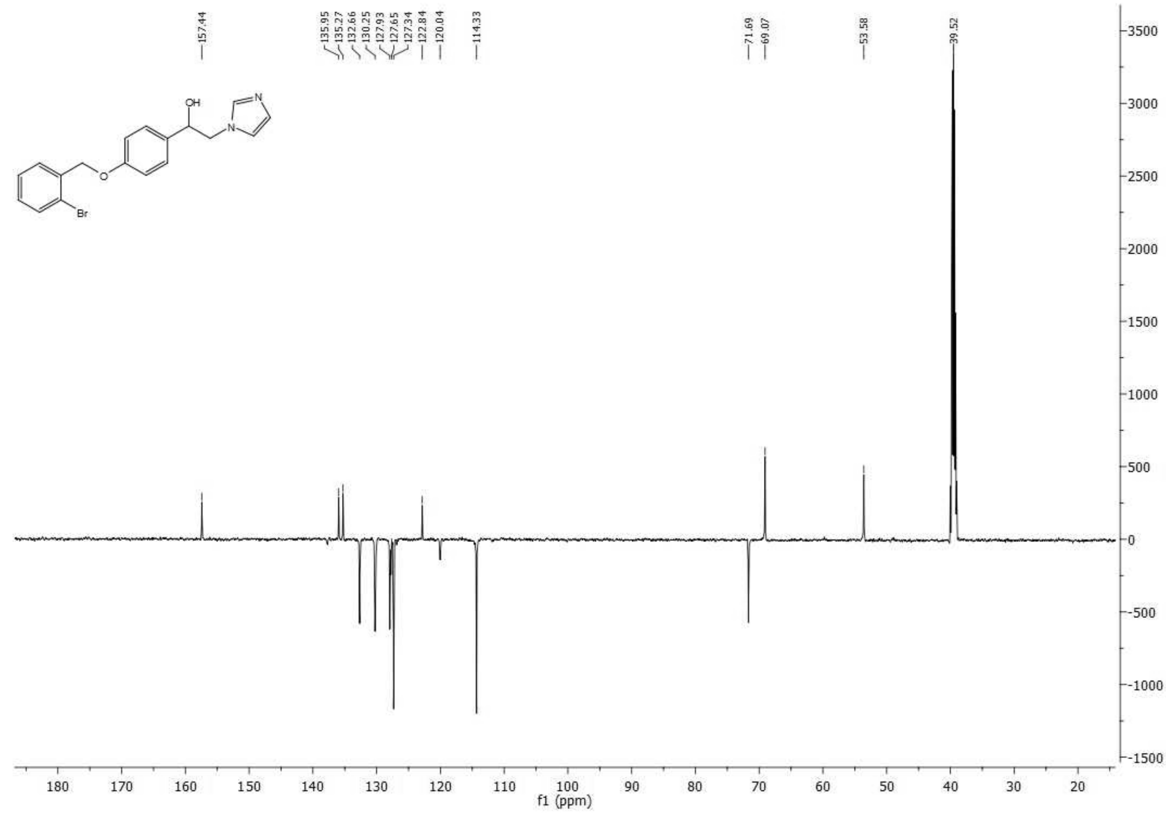

Figure S14. ^{13}C NMR of compound **5d.**

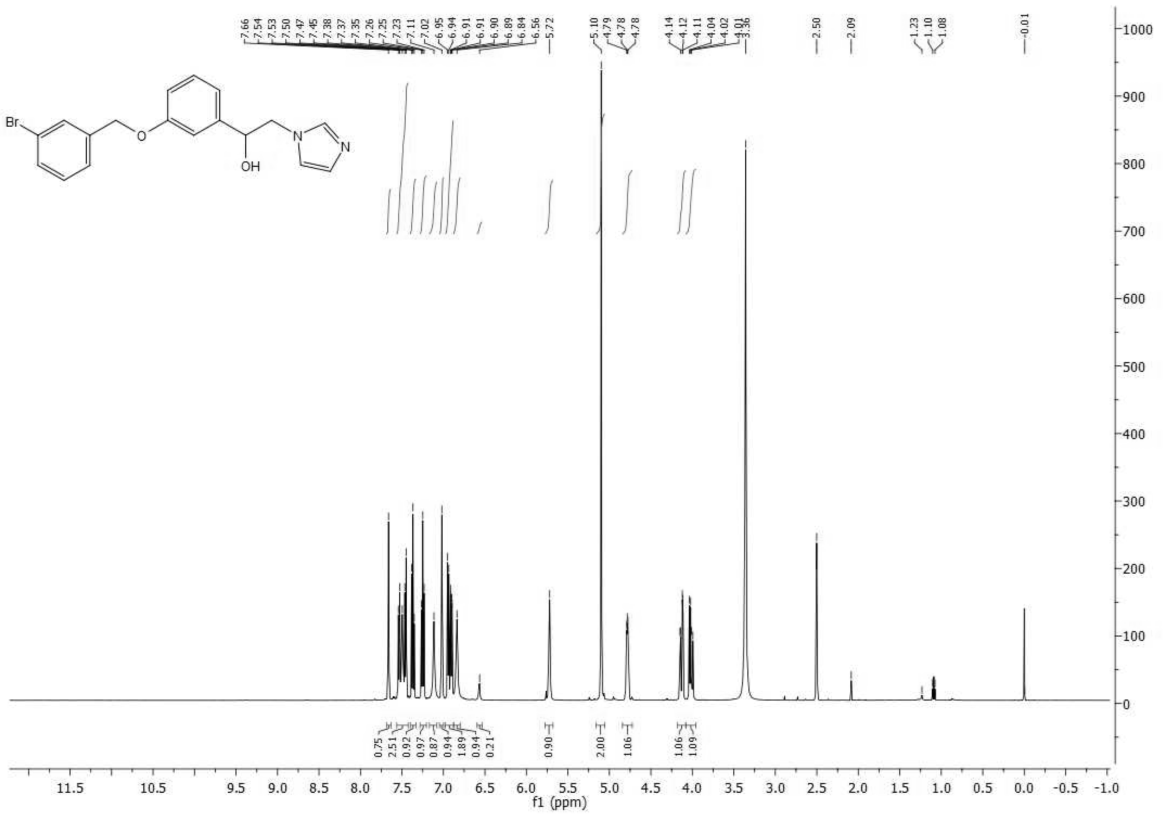

Figure S15. ^{1}H NMR of compound **5e.**

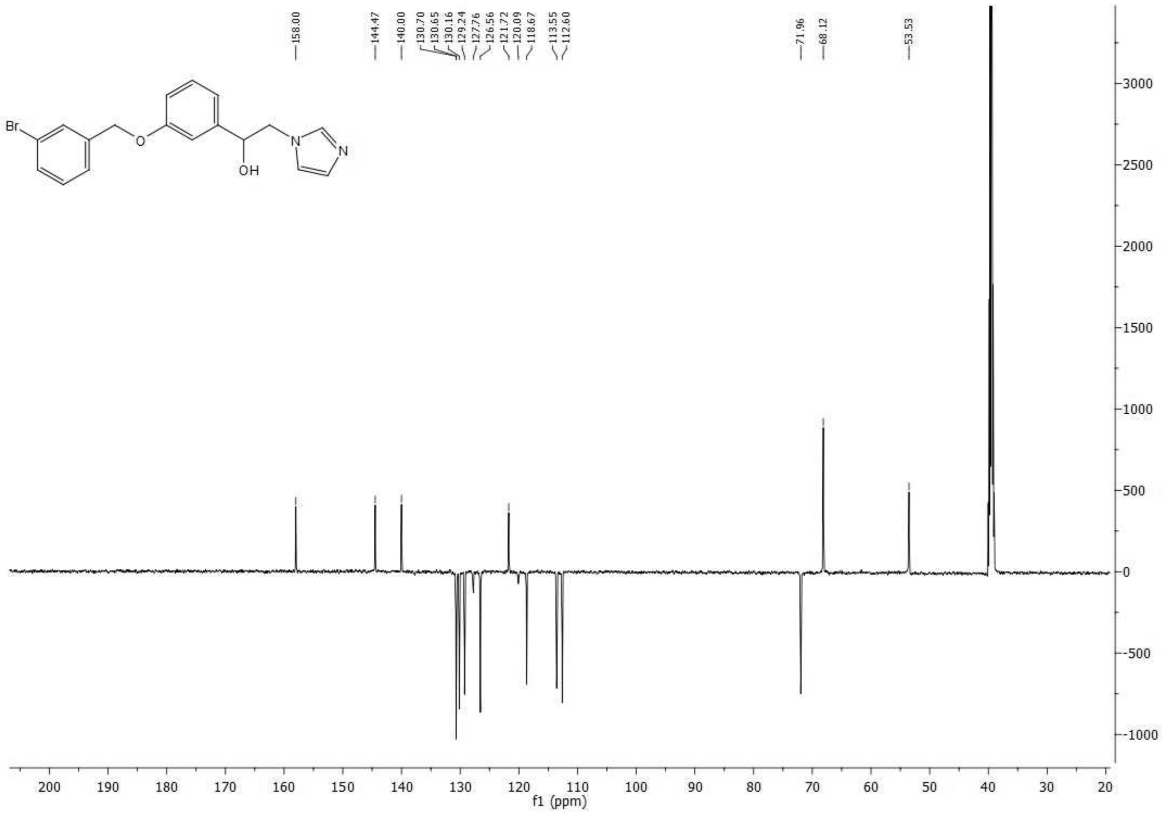

Figure S16. ^{13}C NMR of compound **5e.**

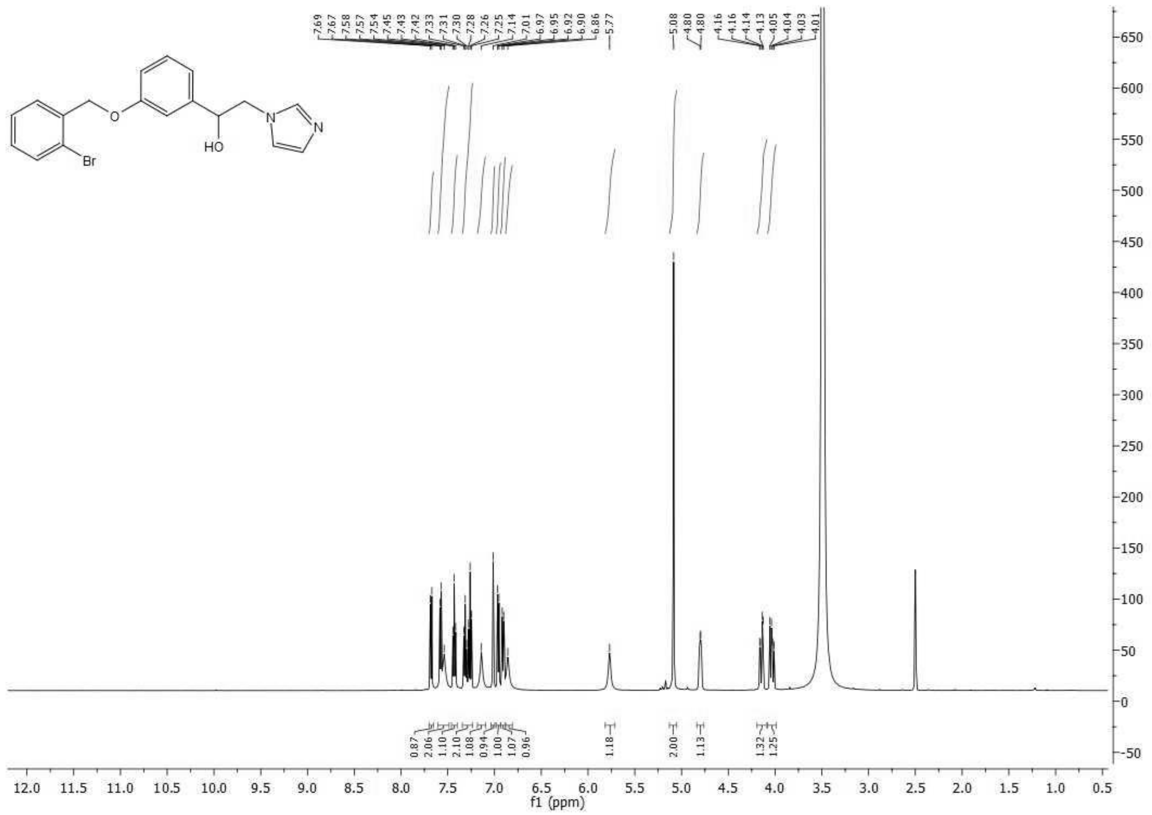

Figure S17. ^{1}H NMR of compound **5f.**

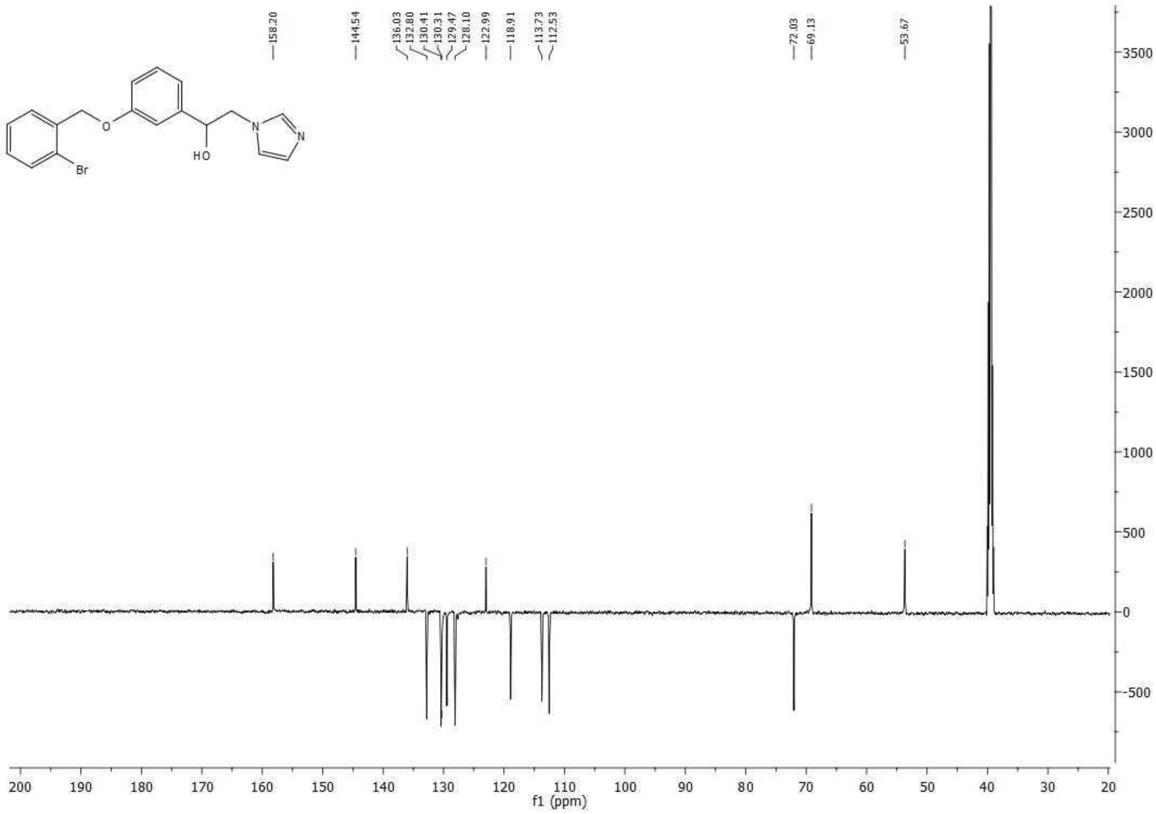

Figure S18. ^{13}C NMR of compound **5f.**

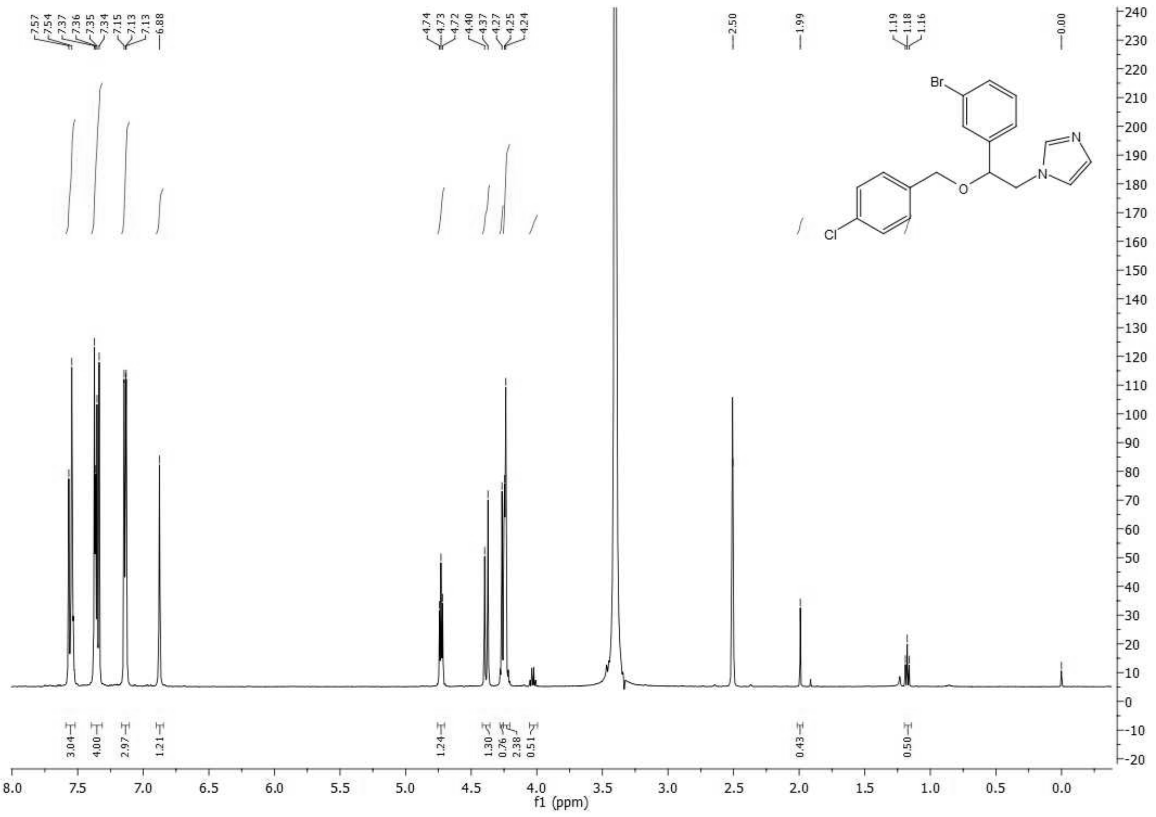

Figure S19. ^{1}H NMR of compound **6a.**

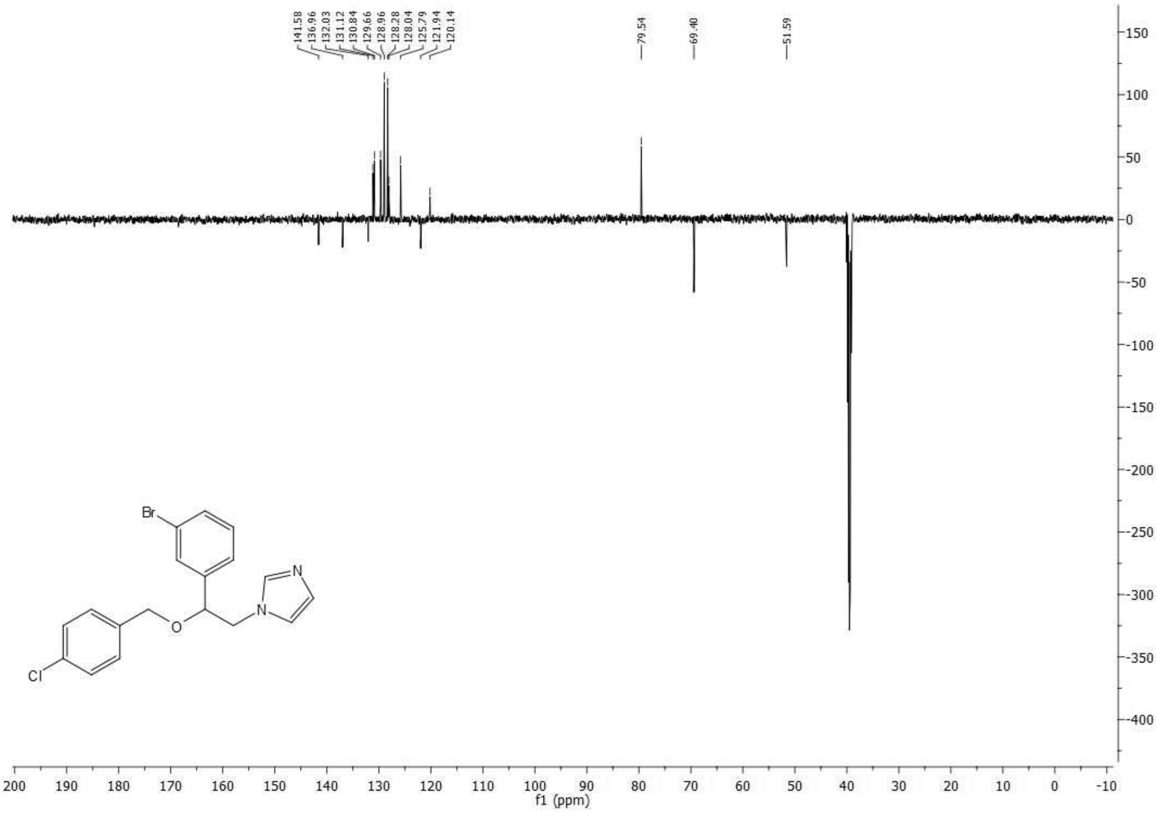

Figure S20. ^{13}C NMR of compound **6a.**

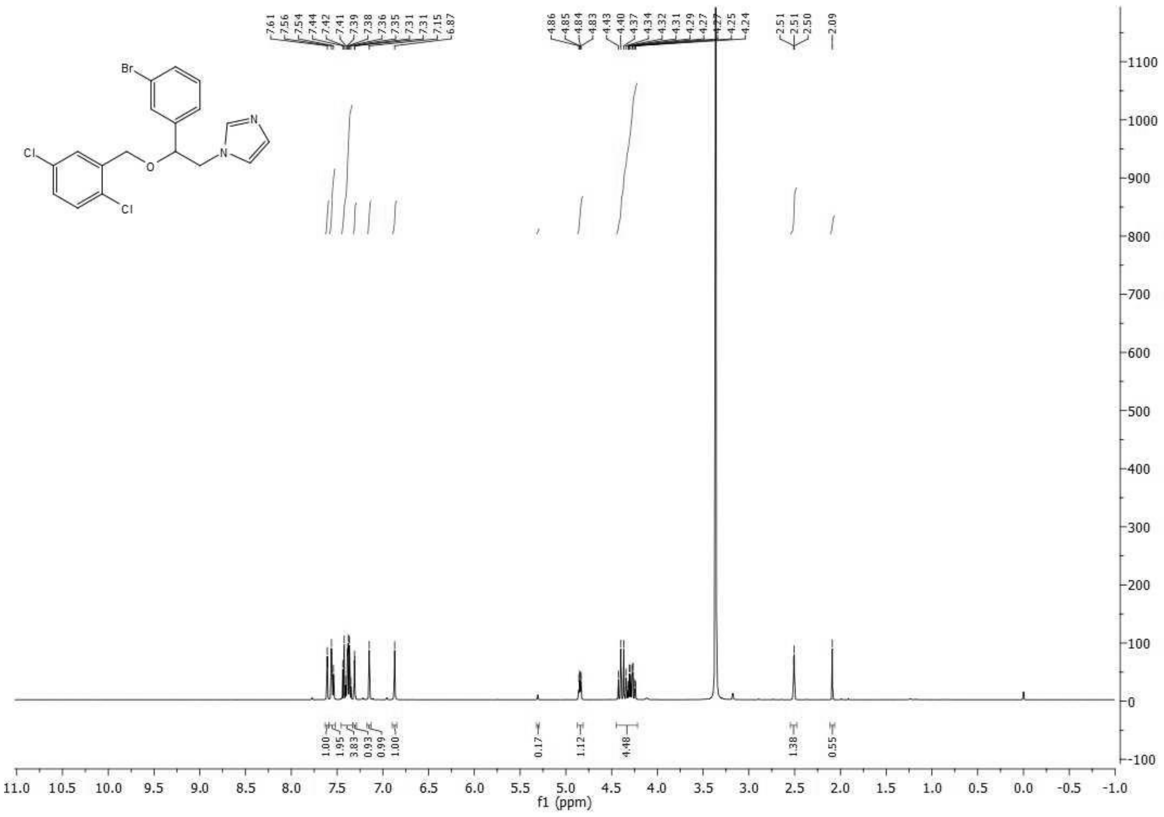

Figure S21. ¹H NMR of compound **6b.**

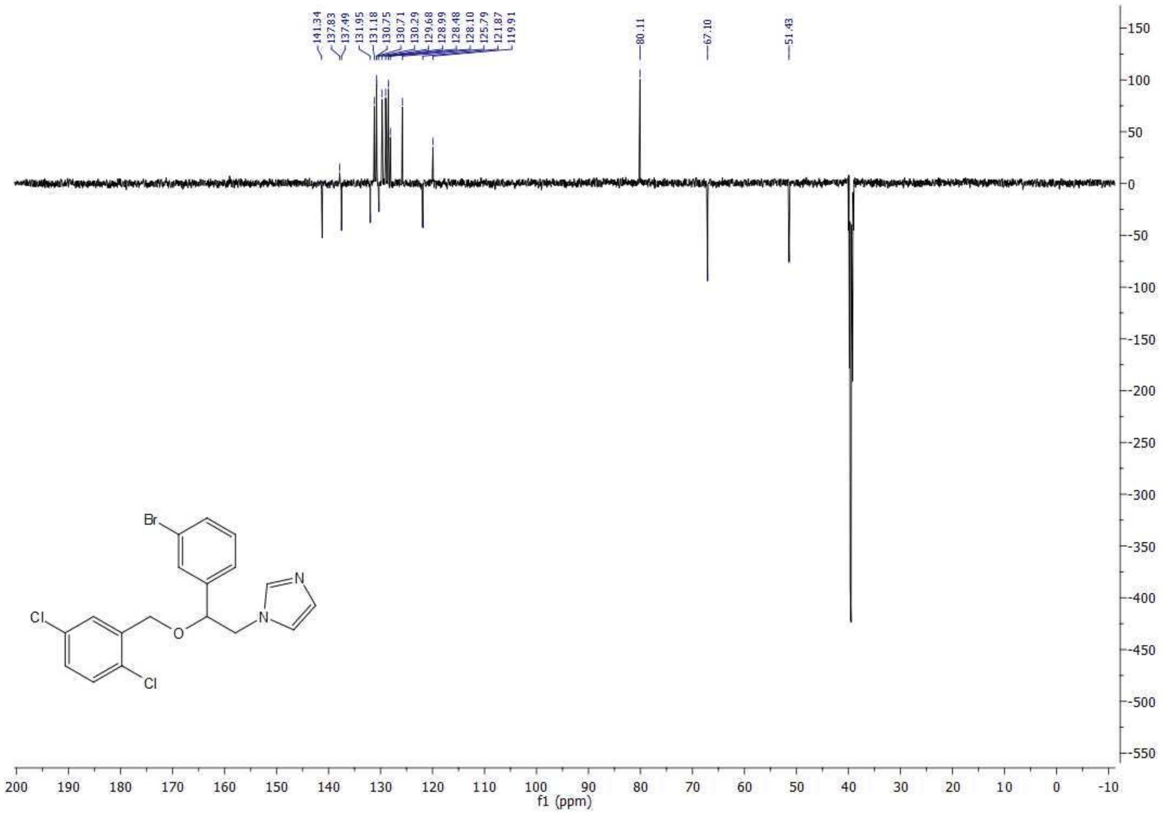

Figure S22. ¹³C NMR of compound **6b.**

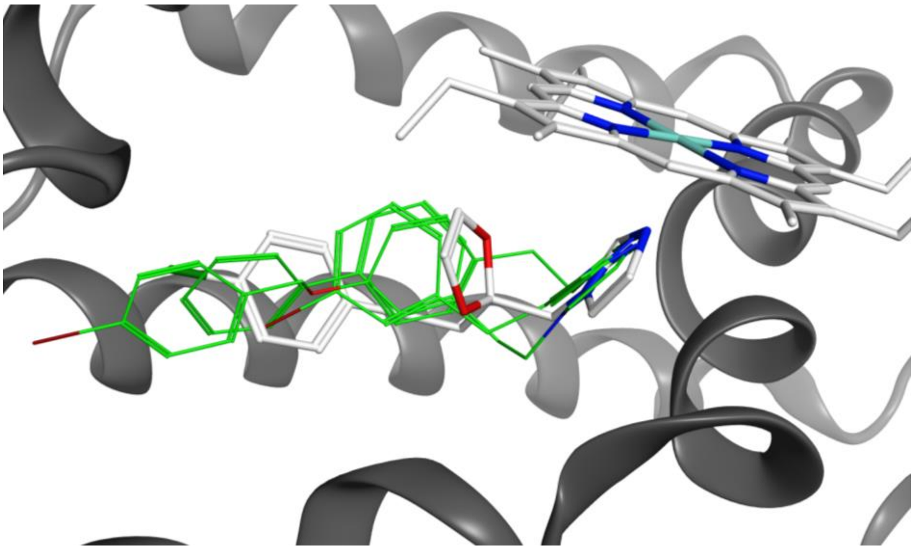

Figure S23. Binding poses of molecules **2a–c** (green) compared to the crystallized pose of QC-15 (white).

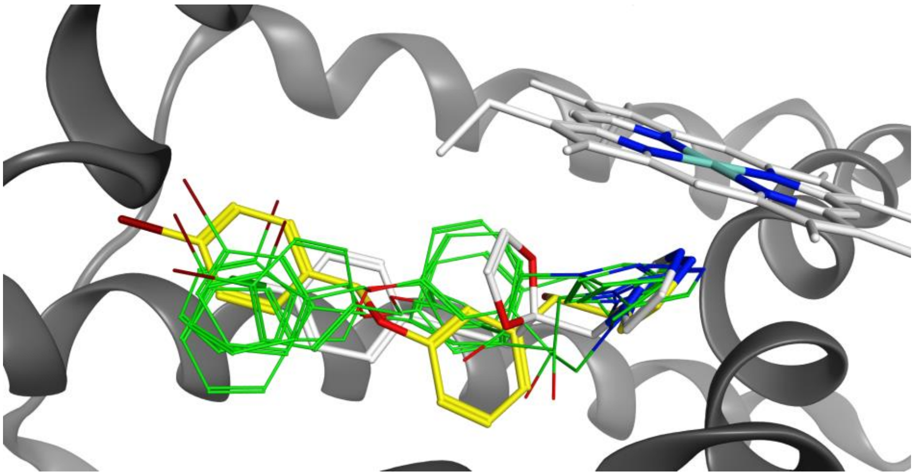

Figure S24. Binding poses of molecules **5b**–**f** (green) compared to the crystallized pose of QC-15 (white). In yellow the binding pose of the most potent compound **5a**.

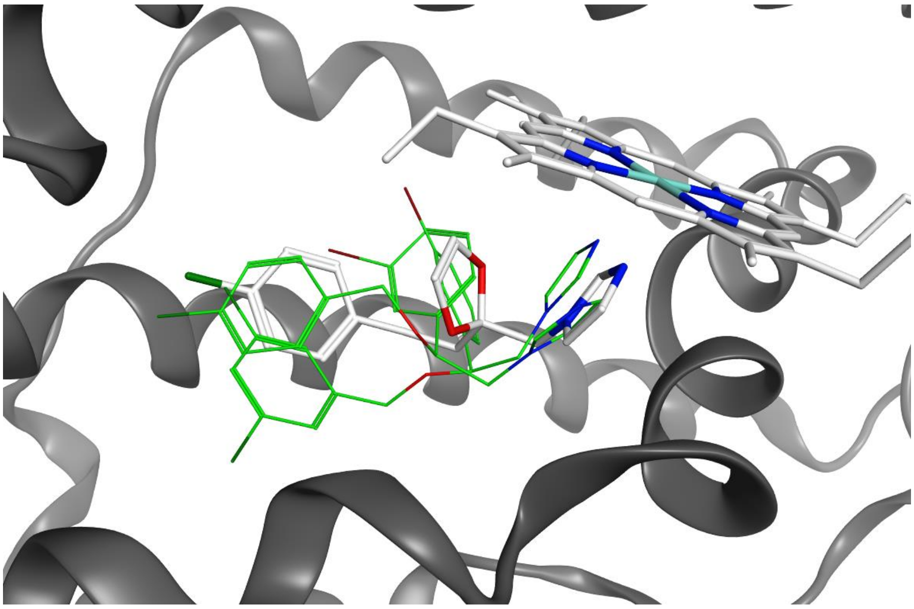

Figure S25. Binding poses of molecules **6a, b** (green) compared to the crystallized pose of QC-15 (white).

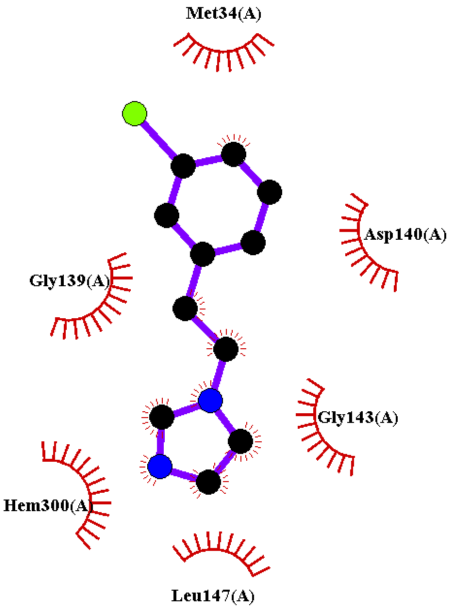

Figure S26. 2D representations of HO-1-molecule **2a** complex.

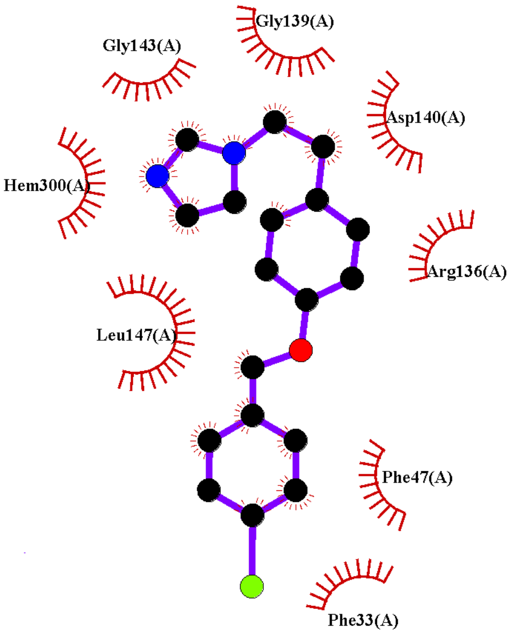

Figure S7. 2D representations of HO-1-molecule **2b** complex.

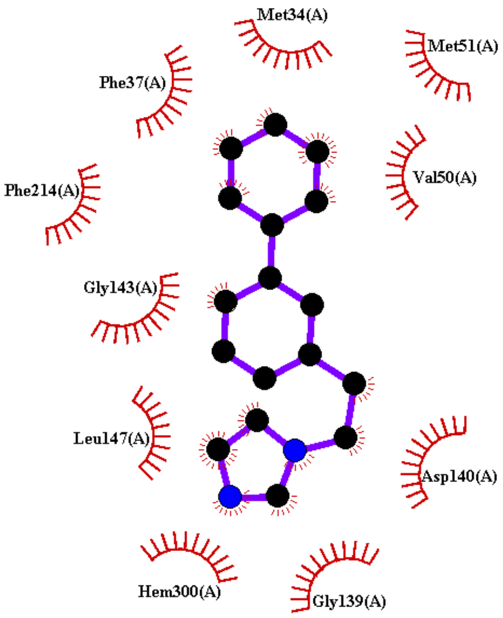

Figure S28. 2D representations of HO-1-molecule **2c** complex.

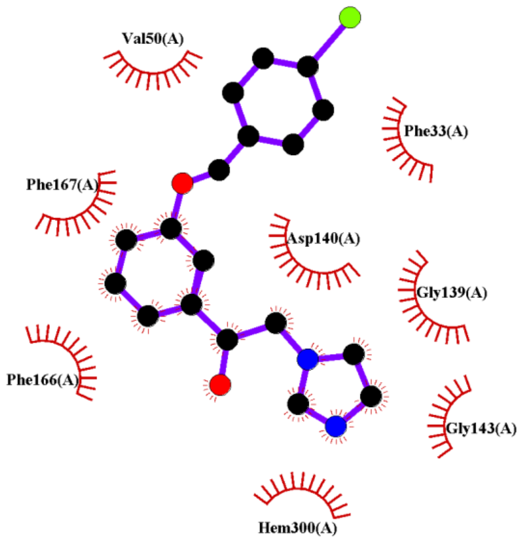

Figure S29. 2D representations of HO-1-molecule **5a** complex.

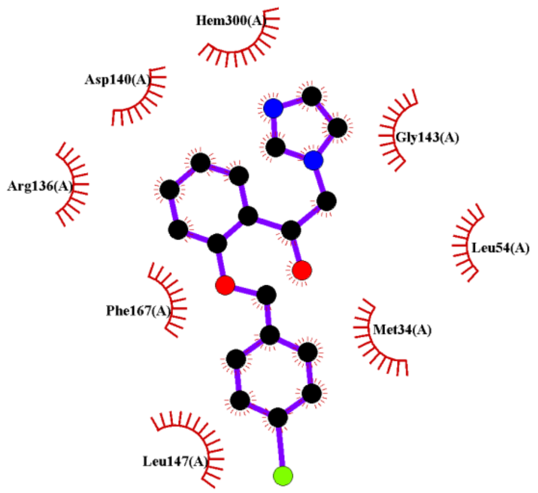

Figure S30. 2D representations of HO-1-molecule **5b** complex.

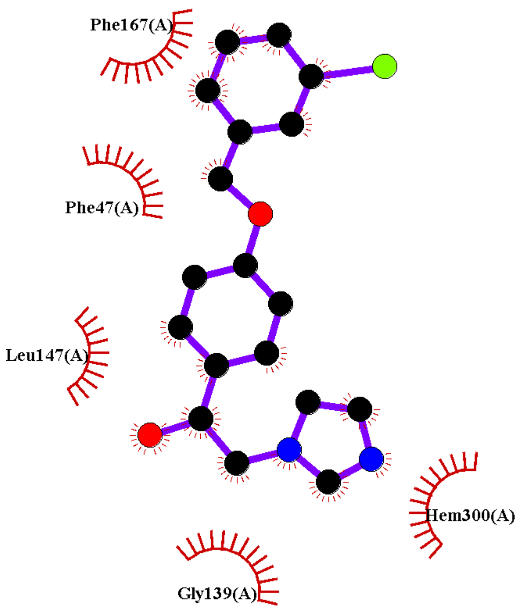

Figure S31. 2D representations of HO-1-molecule **5c** complex.

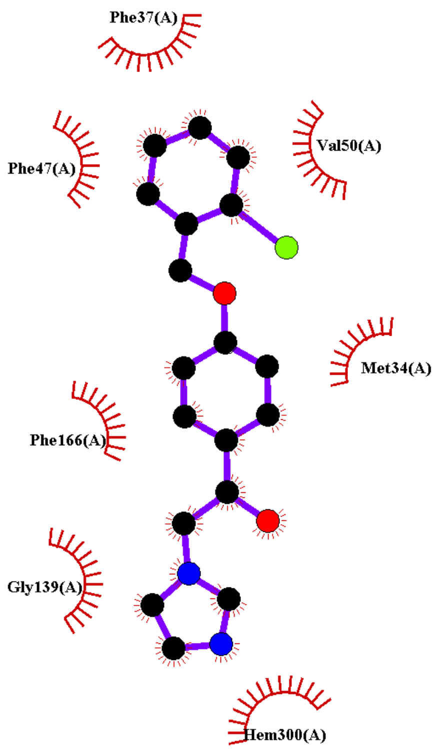

Figure S32. 2D representations of HO-1-molecule **5d** complex.

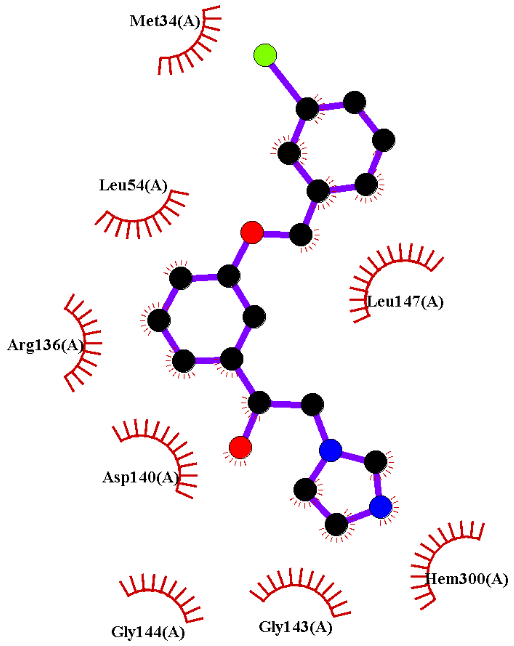

Figure S33. 2D representations of HO-1-molecule **5e** complex.

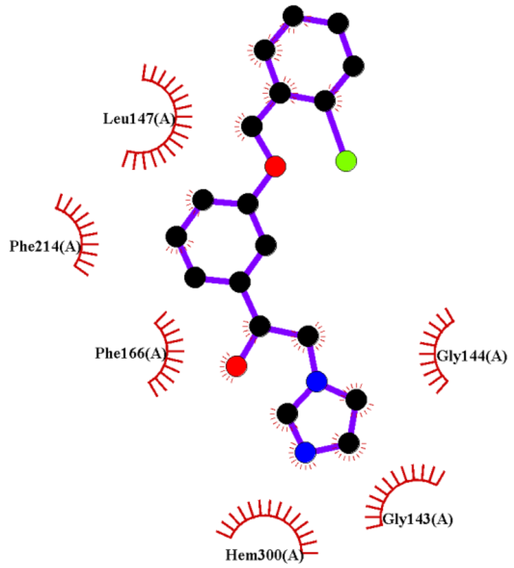

Figure S34. 2D representations of HO-1-molecule **5f** complex.

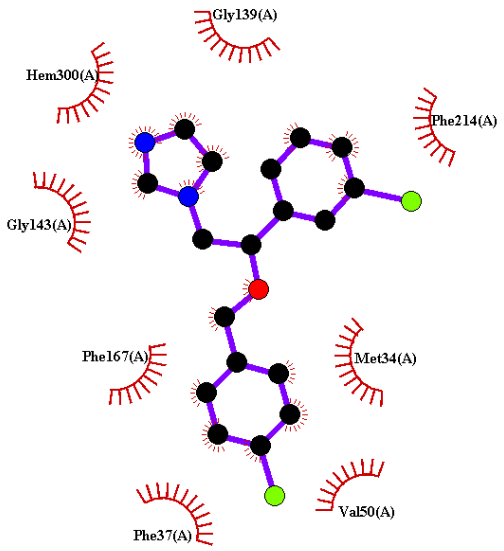

Figure S35. 2D representations of HO-1-molecule **6a** complex.

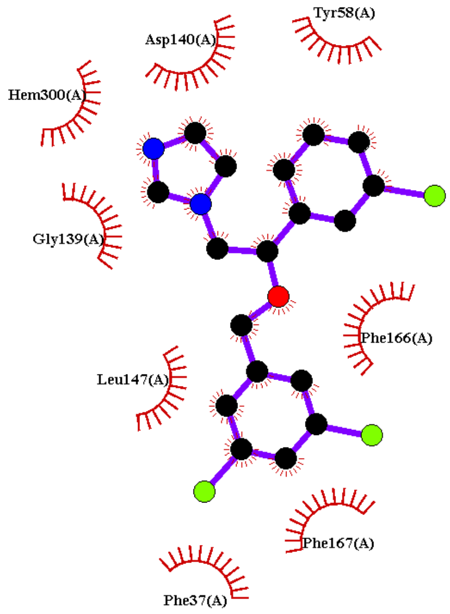

Figure S36. 2D representations of HO-1-molecule **6b** complex.
